# Cell-Penetrating CEBPB and CEBPD Leucine Zipper Decoys as Broadly Acting Anti-Cancer Agents

**DOI:** 10.3390/cancers13102504

**Published:** 2021-05-20

**Authors:** Qing Zhou, Xiotian Sun, Nicolas Pasquier, Parvaneh Jefferson, Trang T. T. Nguyen, Markus D. Siegelin, James M. Angelastro, Lloyd A. Greene

**Affiliations:** 1 Department of Pathology and Cell Biology, Vagelos College of Physicians and Surgeons, Columbia University, New York, NY 10032, USA; qz2266@cumc.columbia.edu (Q.Z.); xs2165@cumc.columbia.edu (X.S.); nicolas.pasquier@utu.fi (N.P.); pjefferson@vassar.edu (P.J.); tn2387@cumc.columbia.edu (T.T.T.N.); ms4169@cumc.columbia.edu (M.D.S.); 2 Department of Molecular Biosciences, School of Veterinary Medicine, University of California Davis, Davis, CA 95616, USA; jmangelastro@ucdavis.edu

**Keywords:** transcription factor, CEBPB, CEBPD, ATF5, decoy

## Abstract

**Simple Summary:**

The gene-regulatory factors ATF5, CEBPB and CEBPD promote survival, growth, metastasis and treatment resistance of a range of cancer cell types. Presently, no drugs target all three at once. Here, with the aim of treating cancers, we designed novel cell-penetrating peptides that interact with and inactivate all three. The peptides Bpep and Dpep kill a range of cancer cell types in culture and in animals. In animals with tumors, they also significantly increase survival time. In contrast, they do not affect survival of non-cancer cells and have no apparent side effects in animals. The peptides work in combination with other anti-cancer treatments. Mechanism studies of how the peptides kill cancer cells indicate a decrease in survival proteins and increase in death proteins. These studies support the potential of Bpep and Dpep as novel, safe agents for the treatment of a variety of cancer types, both as mono- and combination therapies.

**Abstract:**

Transcription factors are key players underlying cancer formation, growth, survival, metastasis and treatment resistance, yet few drugs exist to directly target them. Here, we characterized the in vitro and in vivo anti-cancer efficacy of novel synthetic cell-penetrating peptides (Bpep and Dpep) designed to interfere with the formation of active leucine-zipper-based dimers by CEBPB and CEBPD, transcription factors implicated in multiple malignancies. Both peptides similarly promoted apoptosis of multiple tumor lines of varying origins, without such effects on non-transformed cells. Combined with other treatments (radiation, Taxol, chloroquine, doxorubicin), the peptides acted additively to synergistically and were fully active on Taxol-resistant cells. The peptides suppressed expression of known direct CEBPB/CEBPD targets *IL6*, *IL8* and asparagine synthetase (*ASNS*), supporting their inhibition of transcriptional activation. Mechanisms by which the peptides trigger apoptosis included depletion of pro-survival survivin and a required elevation of pro-apoptotic BMF. Bpep and Dpep significantly slowed tumor growth in mouse models without evident side effects. Dpep significantly prolonged survival in xenograft models. These findings indicate the efficacy and potential of Bpep and Dpep as novel agents to treat a variety of cancers as mono- or combination therapies.

## 1. Introduction

A copious amount of literature links basic leucine zipper transcription factors, ATF5, CEBPB and CEBPD, to tumor formation, growth, metastasis and treatment resistance in a variety of cancers [1,2,3,4,5,6,7,8,9,10,11,12,13,14,15,16,17,18,19,20,21,22,23,24,25,26,27,28,29]. Nevertheless, as with many other transcription factors, few options exist to successfully directly target them for therapeutic purposes.

In the past, we developed the strategy of generating an inducible or cell-penetrating dominant-negative (dn) peptide form of ATF5 for cancer treatment [2,4,30,31,32,33]. This exploited the requirement of leucine zipper proteins to form homo- and/or heterodimers to regulate transcription [34,35,36]. In this context, dn peptides containing specified leucine zipper sequences, but lacking DNA-binding capacity, associated with obligate dimerization partners to form inactive species, thereby effectively sequestering them and interfering with function [33,36]. To permit such dn peptides to enter cells and pass tissue barriers, we fused them with a penetratin domain [31,37]. The resulting cell-penetrating dn peptide for ATF5, CP-dn-ATF5, inhibits survival/growth of a wide variety of cultured tumor cell types and blocks or slows tumor growth and prolongs survival in multiple mouse cancer models [31,32,33]. Pull-down/mass spectrometry studies designed to uncover dn-ATF5′s binding targets have revealed its association with CEBPB and CEBPD, but not with ATF5 itself [33]. Such findings suggest that alternative means of interfering with CEBPB and CEBPD function as well as that of ATF5 would be of potential therapeutic value for cancer treatment.

CEBPB and CEBPD form homodimers and heterodimerize with one another via their leucine zippers [34,35,36]. Our interaction studies indicate that unlike ATF5 itself, they also form leucine-zipper-dependent heterodimers with ATF5 [33]. Taken together, these considerations suggest that leucine-zipper-based dn-forms of CEBPB and CEBPD would not only directly interfere with CEBPB and CEBPD signaling, but also (unlike dn-ATF5) with ATF5 signaling as well. This prompted us to design and assess cell-penetrating dn decoy forms of CEBPB and CEBPD in the context of cancer treatment. Here, we have examined their in vitro and in vivo actions on multiple cancer types, selectivity for killing transformed cells, effects on cancer cell migration, capacity to act in combination with other cancer therapeutics, interference with transcription of known direct CEBPB, CEBPD and ATF5 targets and distal mechanisms of action by which they kill malignant cells.

## 2. Materials and Methods

### 2.1. Cell Culture, Peptides and siRNA Treatment

T98G, LN229, U251, MDA-MB-231, MCF7, MDA-MB-468, A549, HCT116, A375, MeWo, HEK293T and B16-F10 cells were from, and authenticated by, the ATCC. T98G Taxol-resistant cells were established by continuous exposure to incremental Taxol concentrations up to 1 nM. Mgpp3 cells were the kind gift of Dr. Peter Canoll, Columbia University [38]. Cells were grown in DMEM supplemented with 10% FBS and 100 U/mL Penicillin–Streptomycin. HAP1 WT and ATF5KO-1 cells were from Horizon Discovery and authenticated and grown as previously described [33]. Human astrocytes and astrocyte growth medium were from Sciencell Research Laboratories and authenticated by the supplier. MCF10A and HIEC-6 cells were gifts of Drs. Eileen Connolly and Alejandro Chavez (Columbia University, New York, NY, USA), respectively, and cultured and authenticated as described previously [39]. Unless otherwise specified, for experiments concerning cell number, survival or protein/mRNA expression, cells were seeded into 48- or 6-well tissue culture plates precoated with a 0.1 µg/µL poly-D-lysine solution overnight and then air-dried for 15 min.

Bpep, Dpep and mutated peptides were purchased as acetate salts either from CSBio or AlanScientific with the following sequences:

Bpep: RQIKIWFQNRRMKWKKLETQHKVLELTAENERLQKKVEQLSRELSTLRNLFKQL

Dpep: RQIKIWFQNRRMKWKKLVELSAENEKLHQRVEQLTRDLAGLRQFFK

Dpep-mut: RQIKIWFQNRRMKWKKLVEGSAENEKGHQRVEQGTRDGAGGRQFFK

The peptides were dissolved in 10% glycerol in PBS, pH 7.2 and stored as 2 mM aliquots at −80 °C prior to final dilution for experiments.

siRNA: Silencer™ Select (Invitrogen): Negative Control No. 2 siRNA (439084), BMF siRNA s40386 (#4392420), or BMF siRNA s40387 (#4392420) were transfected into 50% confluent cultures using Oligofectamine™ (Invitrogen, Carlsbad, CA, USA) following the supplier’s protocol.

### 2.2. Cell Viability

Cells were seeded in 48-well plates at 1 × 10^4^/well in 0.2 mL of growth medium. Following overnight incubation, cells were refreshed with DMEM with 2% FBS and the indicated concentrations of Bpep or Dpep and other indicated additives. Cell viability was assessed after 6 days or as indicated by cell counting using either a hemocytometer or a Countess II automated cell counter (Life Technologies, Carlsbad, CA, USA). Assays were performed in triplicate.

### 2.3. Colony Formation Assays

Cells were seeded in 6-well plates (200 cells/well) and treated with indicated doses of Bpep or Dpep for 12 days. At the treatment end, colonies were fixed, stained with 0.5% crystal violet and 25% methanol in PBS for 30 min, washed with PBS and quantified. Colonies were defined as containing at least 50 cells. Soft agar colony-forming assays were established in 6-well plates as described [40]. 5 × 10^3^ cells were seeded with specified concentrations of Bpep or Dpep and indicated additives. After 12 days, total colonies were counted.

### 2.4. Plasmids

Fragments encoding wild-type and mutant DN-CEBPB or DN-CEBPD were subcloned into the 5′-BamHI site and the 3′-XhoI site of pCMV-3Tag-2A (Agilent Technologies) plasmid for in-frame 3xMyc-tagged expression. The sequences of the fragments were:

pCMV-3Tag2A-DN-CREBP/B:

MEQKLISEEDLEQKLISEEDLEQKLISEEDLARAGSMASMTGGQQMGRDPDLETQHKVLELTAENERLQKKVEQLSRELSTLRNLFKQL

pCMV-3Tag2A-DN-CEBP/D:

MEQKLISEEDLEQKLISEEDLEQKLISEEDLARAGSMASMTGGQQMGRDPDLVELSAENEKLHQRVEQLTRDLAGLRQFFK

pCMV-3Tag2A-DN-CREBP/B-mut:

MEQKLISEEDLEQKLISEEDLEQKLISEEDLARAGSMASMTGGQQMGRDPDGETQHKVGEGTAENERGQKKVEQGSREGSTGRNGFKQG

pCMV-3Tag2A-DN-CEBP/D-mut:

MEQKLISEEDLEQKLISEEDLEQKLISEEDLARAGSMASMTGGQQMGRDPDGVEGSAENEKGHQRVEQGTRDGAGGRQFFK

### 2.5. Transfection Assays of WT and Mutant DN-CEBPB or DN-CEBPD

Cells were seeded in 48-well plates in triplicate overnight to reach 70–90% confluency. Cultures were transfected with 0.2 µg DNA/well using Lipofectamine™ 3000 (Invitrogen) following the supplier’s protocols. 48 h later, cells were fixed in 4% PFA for 10 min, treated with 0.3% Triton-X for 5 min and blocked with Superblock (Thermo Fisher, Rockford, IL, USA) for 30 min at room temperature. Cells were then incubated with mouse anti-myc (Cell Signaling Technology, Danvers, MA, USA) for 1 h, washed with PBS and incubated with the secondary antibody for another hour. Cultures were stained with Hoechst 33342 to detect total cells and apoptotic nuclei. Images were acquired using a Zeiss epifluorescence microscope with a digital camera and Axiovision software. The proportion of cells with apoptotic nuclei was quantified by scoring all transfected cells from 4 random fields for each sample.

### 2.6. Annexin V-FITC/PI Double Staining Assay

Apoptosis was evaluated using the FITC Annexin V Apoptosis Detection Kit I (BD Biosciences) using the supplier’s protocol, followed by analysis in the Columbia HICCC flow Cytometry Core.

### 2.7. qPCR

Cells were seeded in 6-well plates, treated with 20 µM Bpep or Dpep for 48 h and lysed. Total RNA purification, cDNA synthesis and qPCR were carried out as previously described [33]. Primer pairs were:

18S ribosomal RNA Forward primer: 5′-AGTCCCTGCCCTTTGTACACA-3′

18S ribosomal RNA Reverse primer: 5′-GATCCGAGGGCCTCACTAAAC-3′

IL6 Forward primer: 5′-CCAGAGCTGTGCAGATGAGTA-3′

IL6 Reverse primer: 5′-TGGGTCAGGGGTGGTTATTG-3′

IL8 Forward primer: 5′-CTCTTGGCAGCCTTCCTGATT-3′

IL8 Reverse primer: 5′-TATGCACTGACATCTAAGTTCTTTAGC-3′

BMF Forward primer: 5′-CCACCAGCCAGGAAGACAAAG-3′

BMF Reverse primer: 5′-TGCTCCCCAATGGGCAAGACT-3′

ASNS Forward primer: 5′-CTGTGAAGAACAACCTCAGGATC-3′

ASNS Reverse primer: 5′-AACAGAGTGGCAGCAACCAAGC-3′

### 2.8. Western Immunoblotting

Cells were seeded into 6-well dishes, treated with 20 µM Bpep or Dpep for 72 h and processed for Western immunoblotting and imaging as previously described [33]. Antibodies used were: rabbit anti-survivin (Cell Signaling Technology, Danvers, MA, USA #2808), rabbit anti-MCL-1 (Cell Signaling Technology, Danvers, MA, USA #94296s), rabbit anti-BCL-2 (Cell Signaling Technology, Danvers, MA, USA #15071s) and mouse anti-ACTIN (Cell Signaling Technology, Danvers, MA, USA #3700).

### 2.9. Migration Scratch Assay

Cells were seeded in 6-well plates to form monolayers and were either pretreated or not with 20 µM Bpep or Dpep for 20 h. Scratches were made with a 200 μL pipette tip. Cells were then treated for another 20 h. Images were taken at 0 and 20 h for determination of scratch widths.

### 2.10. Mouse Tumor Models

Animal procedures were approved by the Columbia University IACUC (protocol AC-AABE2555) and followed guidelines set forth by the National Institutes of Health guide for care and use of laboratory animals. 1 × 10^6^ A375 HCT116 or B16-F10 cells were implanted subcutaneously into flanks of 6–8 week-old female NCR nude mice (Taconic, Rensselaer, NY, USA) or C57BL/6 mice (Taconic, Rensselaer, NY, USA), respectively. Each mouse received three implantations. When tumors reached 100–200 mm^3^, mice were randomized into different groups and treated intraperitoneally with 100 µL/mouse of vehicle or peptides 3–4 times/week as indicated. The amount of peptide per treatment ranged between 10–50 mg/kg as indicated in Figures and Figure legends. Tumor sizes were measured with calipers and calculated as (length × width^2^)/2. Body weights were monitored at each time point. For survival studies, mice were sacrificed once a single tumor reached a preset endpoint of 1000 mm^3^. H&E and TUNEL staining of harvested tumors were conducted by the Columbia University Molecular Pathology facility.

### 2.11. Statistical Analyses

All experiments were performed at least in triplicate. Data are expressed as mean ± SEM. Statistical significance was calculated using a two-tailed Student’s *t*-test or ANOVA (for multiple comparisons). IC_50_ values were calculated by non-linear regression.

## 3. Results

### 3.1. dn-CEBPB and -CEBPD Expression Compromises Cancer Cell Survival

**Figure 1:**
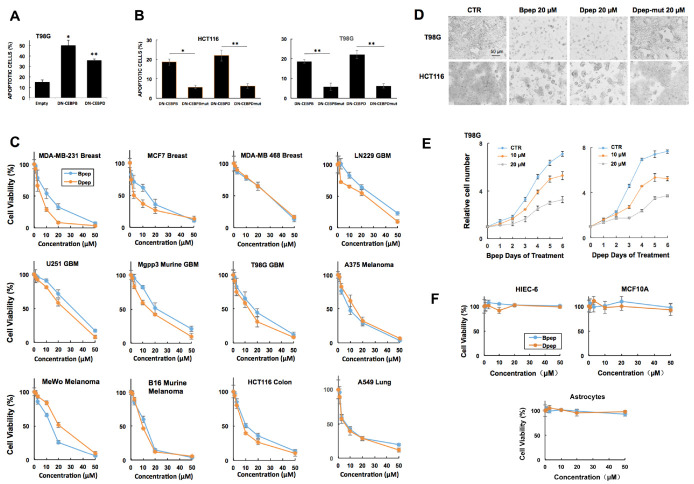
DN-CEBPB, DN-CEBPD, Bpep and Dpep suppressed growth/survival of multiple cancer cell lines of different origins. (**A**). Transfection with plasmids expressing DN-CEBPB and DN-CEBPD suppressed growth/survival of T98G glioblastoma cells. Cells were transfected with indicated plasmids and 3 days later, assessed for the proportion of transfected (myc-tag expressing) cells with apoptotic nuclei. In these and the following panels, values are means, with error bars representing ± SEM. *p* values (vs. empty vector control) are represented here and in the following Figures as follows: * *p* < 0.05, ** *p* < 0.005, *** *p* < 0.0005 (*n* = 3). Comparable results were achieved in 3 additional independent experiments. (**B**). The efficacy of DN-CEBPB and DN-CEBPD in suppressing the growth/survival of tumor cells required an intact leucine zipper. Cells were transfected with the indicated plasmids expressing DN-CEBPB and DN-CEBPD as well as corresponding forms DN-CEBPBmut and DN-CEBPDmut, in which the heptad repeat leucines were replaced with glycine residues. Two days later, the proportion of apoptotic cells was determined as in A. The experiment was carried out with 5 replicate cultures per condition. *p* values are given vs. corresponding mut controls. (**C**). Bpep and Dpep suppressed growth/survival of multiple cancer cell lines of various origins in a dose-dependent manner. Cultures were exposed to the indicated concentrations of peptides for 6 days and then assessed for relative cell numbers. Graphs represent single experiments carried out in triplicate. Comparable results were achieved in 1 or more additional independent experiments per line. (**D**). Images of tumor cell cultures exposed to the vehicle (CTR = Control), 20 µM Bpep, Dpep or Dpep-mut for 6 days. (**E**). Time courses of relative cell numbers in T98G cultures exposed to 0, 10 or 20 µM Bpep or Dpep. Peptides were applied as a single dose on day 0. Data are from one of three independent experiments carried out in triplicate, each with comparable results. (**F**). The lack of effect of Bpep and Dpep on non-transformed cells. Replicate cultures of indicated non-transformed cell types were exposed to indicated concentrations of Bpep and Dpep for 6 days and assessed for cell numbers. Data are from one of two independent experiments, each in triplicate with comparable results.

To test the hypothesis that dn-decoy forms of CEBPB and CEBPD kill cancer cells, we transfected T98G glioblastoma cells with vectors expressing myc-tagged CEBPB or CEBPD leucine zipper sequences, or tag alone. Assessment of transfected cell nuclei after 3 days revealed that both dn constructs caused significant apoptosis compared with the control (Figure 1A). As an additional control, we transfected T98G and HCT116 colon cancer cells with the constructs as well as with constructs in which the heptad repeat leucines in the CEBPB and CEBPD leucine zippers were replaced with glycines. The dn constructs significantly increased apoptosis compared with the mutant forms, indicating a requirement for the intact leucine zipper as well as an absence of non-specific toxicity (Figure 1B).

### 3.2. Cell-Penetrating dn-CEBPB and dn-CEBPD Peptides Compromise Growth/Survival of Multiple Cancer Cell Lines

We next designed cell-penetrating peptide forms of dn-CEBPB and dn-CEBPD, containing a cell penetrating penetratin domain followed by the leucine zipper sequence of the corresponding proteins. We will henceforth refer to these peptides as Bpep and Dpep, respectively. While the peptides were freely soluble in PBS + 10% glycerol at 2 mM, in a culture medium containing 2% or greater fetal bovine serum, there was minor precipitation at 50 µM that increased with elevated concentrations. Dose-response experiments carried out for 6 days revealed that both peptides inhibited growth/survival of multiple cancer cell lines with 50% efficacy (IC50) generally in the range of 10–30 µM. Assessed cancer types included glioblastoma (T98G, LN229, U251, murine MGPP3), melanoma (A375, MeWo, murine B16), breast (MDA-MB-231, MCF7, MDA-MB-468), lung adenocarcinoma (A549), colon cancer (HCT116), myelogenous leukemia (HAP1) and transformed embryonic kidney cells (293T) (Figure 1C,D; Supplementary Figures S1A and S2A). A time course with T98G cells revealed that peptide effects were detectable by day 1 and continued through 6 days of exposure (Figure 1E).

Dose-responses for Bpep and Dpep were generally similar, but not identical across multiple lines, raising the question of whether the two might act additively or synergistically. Multiple lines were exposed to various concentrations of Bpep or Dpep alone or in a 1:1 combination and assessed for growth/survival. The outcome (Supplementary Figure S1B) indicates the two peptides act near-additively (with calculated Combination Indexes (CI) of 0.91 ± 0.07, 0.9 ± 0.11, 0.93 ± 0.08 and 0.91 ± 0.17, respectively), suggesting they target the same or similar pathways.

Bpep and Dpep were designed to interact with targets by specific leucine-zipper interactions. To confirm the importance of this interaction and to rule out non-specific toxicity, parallel assays were carried out on multiple lines with unmodified peptides and with Dpep mutated to replace heptad repeat leucines in the leucine zipper with glycine residues. The outcome showed a greatly decreased potency of the modified peptides, indicating reliance on the intact leucine zipper for full activity (Figure 1D; Supplementary Figure S1C).

An important question is whether Bpep/Dpep effects on cell survival/growth are selective for cancer cells. To assess this as well as possible non-specific toxicity, non-transformed cells including primary astrocytes, MCF10A breast cells and HIEC-6 intestinal epithelial cells were treated with the peptides. No effects were observed on survival/growth at up to 50 µM for 6 days of treatment, indicating an absence of activity or toxicity on normal cells (Figure 1F, Supplementary Figure S2B). These observations also suggest that the minor activity of the mutated peptides on cancer cells reflects a low level of residual target binding rather than toxicity.

**Figure 2:**
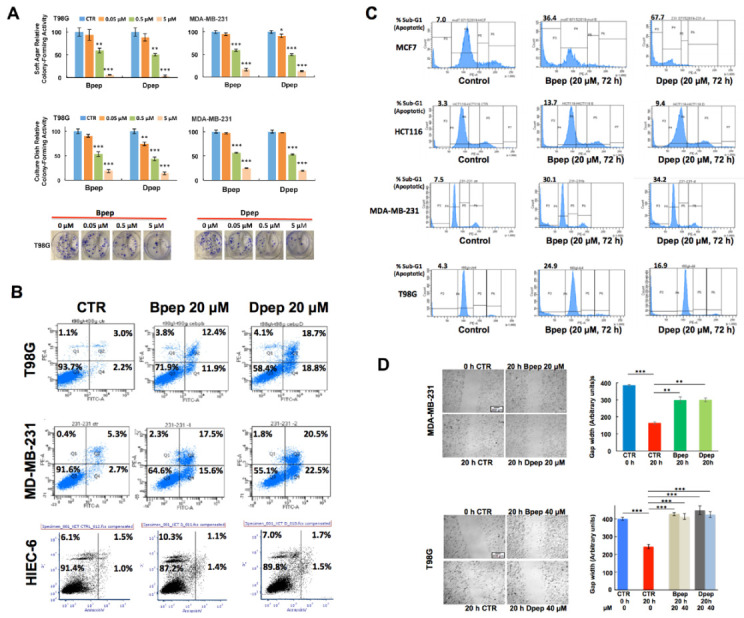
Bpep and Dpep inhibited colony formation, promoted apoptosis of cancer cells and suppressed tumor cell migration. (**A**). Bpep and Dpep suppressed colony formation by multiple cancer cell lines. The upper panel shows relative colony formation on soft agar after 12 days of treatment with indicated concentrations of Bpep or Dpep. *p* values vs. control are indicated as described in the legend of Figure 1. The middle panel shows relative colony formation on culture dishes after 12 days of treatment with indicated concentrations of Bpep or Dpep. The lower panel shows images of colonies growing on culture dishes and treated with indicated concentrations of Bpep or Dpep. The experiment was repeated 3 times in triplicate with comparable results. (**B**). Bpep and Dpep promoted apoptosis of cancer cells, but not of non-transformed cells, as assessed by flow cytometry of Annexin V-FITC/PI-stained cells. Cells were treated with 0 (control, CTR) or 20 µM Bpep or Dpep for 3 days prior to analysis. Numbers indicate the proportion of cells in corresponding quadrants, with early apoptotic and late apoptotic cells in the lower and upper right quadrants, respectively. Data are from one of two independent experiments, each with comparable results. (**C**). Bpep and Dpep promoted apoptosis of multiple cancer cell types as assessed by flow cytometric analysis of sub-G1 DNA levels in PI-stained cultures. Cultures were treated with 0 or 20 µM Bpep or Dpep for 3 days prior to analysis. Values at the upper left of each plot represent % of sub-G1 signal relative to total signal. Data represent results of one experiment. (**D**). Bpep and Dpep inhibited migration of MDA-MB-231 and T98G cells. Replicate cultures in two independent experiments were submitted to scratch assays and then treated for 20 h with indicated concentrations of Bpep or Dpep. Cultures were photographed at the time of scratch application and 20 h after treatment. Representative images show scratch width at the time of scratch and 20 h later under indicated conditions. Graphs in the right panels show relative gap widths at indicated times and the conditions of treatment for each cell line. Peptide concentration in the upper graph is 20 µM. *p* values are indicated vs. 20 h control (*n* = 3–16). * *p* < 0.05, ** *p* < 0.005, *** *p* < 0.0005.

In addition to assessing peptide effects in monolayer cultures, we evaluated their actions in colony-forming assays in soft-agar and on culture dishes, conditions that may more closely model in vivo growth and metastasis. In both models, Bpep and Dpep suppressed colony formation by multiple lines with IC50′s in the 500 nM range and efficacies of 75–97% at 5 µM (Figure 2A, Supplementary Figure S3A).

While experiments with Bpep and Dpep indicated an apoptotic response, we directly evaluated this by flow cytometry of PI/Annexin V-stained cancer lines treated with or without 20 µM Bpep or Dpep for 3 days. This revealed substantial peptide-stimulated increases in apoptosis (Figure 2B; Supplementary Figure S3B). In contrast, treated non-transformed intestinal HIEC-6 epithelial cells showed no increase in apoptosis (Figure 2B). To confirm the apoptotic response of cancer cells, we also performed flow analysis of sub-G1 DNA levels in multiple PI-stained lines with or without 3-days’ treatment with 20 µM Bpep or Dpep. This too showed that the peptides elicit a substantial apoptosis (Figure 2C).

ATF5, CEBPB and CEBPD are reported to promote tumor cell migration and metastasis [11,13,23,25]. To assess whether our peptides would suppress this activity, we performed migration assays in which T98G and MDA-MB-231 cultures were subjected to scratches and assessed 20 h later (a time with little cell death with the peptides) for cell movement into the gap with or without Bpep or Dpep. In both lines, the peptides significantly inhibited migration (Figure 2D). Inhibition was also observed in MDA-MB-231 cultures pretreated with the peptides for 20 h, scratched and assessed for gap size for 20 h (Supplementary Figure S3C).

### 3.3. Bpep and Dpep Act in Combination with Other Cancer Therapeutics

We next assessed Bpep and Dpep actions in combination with multiple presently employed cancer therapeutics. We were particularly interested to learn whether the peptides might interfere with other therapies, or act additively or synergistically with them, and whether they would act on cells resistant to such therapies.

**Figure 3:**
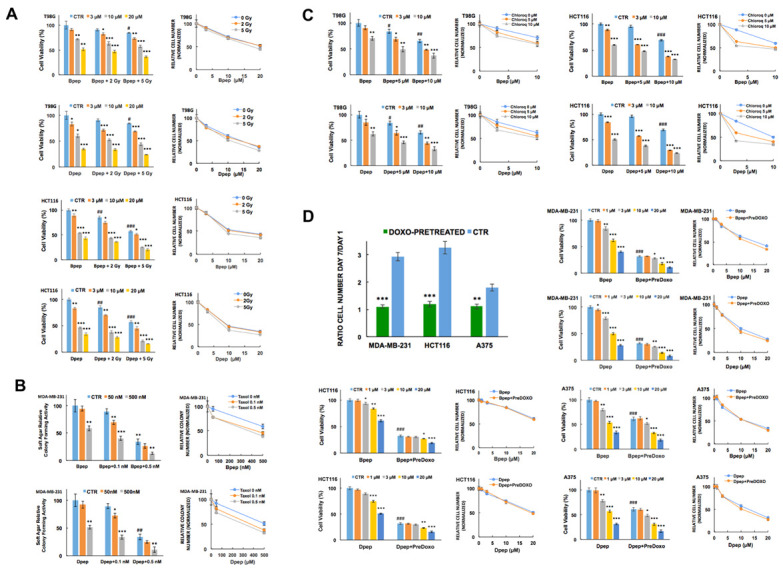
Bpep and Dpep acted additively to synergistically in combination with other anti-cancer treatments and were active on non- or slowly dividing cancer cells. (**A**). Bpep and Dpep acted in combination with gamma radiation. Replicate cultures of T98G and HCT116 cells were subjected to 0, 2 or 5 Gy of gamma radiation and immediately thereafter, treated with 0, 3, 10 or 20 µM Bpep or Dpep for 6 days before assessment of cell numbers. Column graphs on the left show relative (to untreated control cultures) cell numbers under each condition. Line graphs on the right show cell numbers normalized in each case so that 100 represents the relative number of surviving cells that received the indicated level of radiation treatment, but no peptide treatment. Juxtaposition of data points indicates additivity while non-juxtaposition indicates potential synergism or antagonism. The figure represents one of two independent experiments with a similar outcome, each carried out in triplicate. *p* values for radiation treatment vs. controls (all in absence of peptides) are indicated as follows: # *p* < 0.05; ## *p* < 0.005; ### *p* < 0.0005. *p* values for peptide treatment ± irradiation vs. corresponding control are indicated by * as in the legend of Figure 1. (**B**). Bpep and Dpep acted in combination with Taxol on MDA-MB-231 cells as illustrated by colony-forming assays in soft agar. Replicate (*n* = 3) cultures were treated with 0, 0.1 or 0.5 nM Taxol in combination with 0, 50 or 500 nM Bpep or Dpep and assessed 12 days later for relative colony formation in soft agar. Data are shown on the left as relative (to untreated control cultures) colony numbers in each condition and on the right as line graphs normalized as described in panel A. *p* values for Taxol-treated vs. control (all in absence of peptides) are indicated by # as above. *p* values for peptide ± Taxol treatment vs. corresponding controls are indicated by * as in the legend of Figure 1. (**C**). Bpep and Dpep acted in combination with chloroquine. The graph shows data from one of two independent experiments, each in triplicate, with comparable outcomes. T98G and HCT116 cells were cultured for 6 days in the presence of indicated concentrations of Bpep and Dpep and/or chloroquine and assessed for cell numbers. Graphs on the left show relative (to untreated control cultures) cell numbers under each condition, while line graphs on right show cell numbers normalized as described in panel A. *p* values for chloroquine-treated vs. control (all in absence of peptides) are indicated by # as above. *p* values for peptide ± chloroquine treatment vs. corresponding controls are indicated by * as above. (**D**). Bpep and Dpep suppressed survival of non- or slowly dividing cancer cells. Replicate cultures of MDA-MB-231, HCT116 and A375 cells were pretreated for 24 h with or without 100 nM doxorubicin to inhibit proliferation and then for 6 days with the indicated concentrations of Bpep or Dpep. Data are from one experiment in triplicate. The upper left panel shows the ratios of cell numbers at the end of the experiment (day 7) compared with one day after pre-treatment with or without doxorubicin alone (day 1). *p* values for doxorubicin pretreated vs. control are indicated by # as above. The remaining panels show cell survival in cultures pre-treated with or without doxorubicin and/or with or without various concentrations of Bpep or Dpep. Bar graphs represent % survival relative to that observed with no treatment. Corresponding line graphs are normalized so that 100 represents the relative number of surviving cells that were pre-treated with doxorubicin, but no peptide treatment. Near-juxtaposition of data points indicates that cells retained similar sensitivity to peptides irrespective of whether or not they were pre-treated with doxorubicin. *p* values for control vs. doxorubicin pretreatment (all in the absence of peptides) are indicated by # as above. *p* values for peptide ± doxorubicin pre-treatment vs. corresponding controls are indicated by * as above. * *p* < 0.05, ** *p* < 0.005, *** *p* < 0.0005.

ATF5, CEBPB and CEBPD have been reported to contribute to cancer cell radiation resistance [12,14,24]. To assess Bpep and Dpep in combination with radiation treatment, T98G and HCT116 cells (representing cancer types treated with radiotherapy) were either co-exposed to gamma radiation and the peptides, or irradiated and one day later subjected to peptide treatment. In both cases, comparison with untreated and monotherapy-treated cells indicated that the peptides did not preclude response to radiation and (as seen in normalized plots) that the peptides acted approximately additively to somewhat synergistically with radiation to suppress tumor cell growth/survival (co-treatment: T98G, CI = 0.74 ± 0.04 for Bpep, 0.84 ± 0.07 for Dpep; HCT116, CI = 0.93 ± 0.06 for Bpep, 0.92 ± 0.08 for Dpep. Radiation pre-treatment: T98G, CI = 0.61 ± 0.05 for Bpep, 0.68 ± 0.07 for Dpep; HCT116, 0.89 ± 0.04 for Bpep, 0.86 ± 0.05 for Dpep) (Figure 3A; Supplementary Figure S4A). Similar results were achieved with Dpep and T98G cells as determined by colony formation in soft agar (Supplementary Figure S4B). Although there were too few points to calculate a CI value, an apparent synergistic effect was particularly evident in the combination of 5 µM peptide with 5 Gy.

Past studies [10,19,27] have implicated ATF5, CEBPB and CEBPD in the regulation of cancer cell responsiveness to the microtubule-targeting drug Taxol (paclitaxel), which is used to treat breast and other tumor types. We therefore compared responses of MCF7 and triple negative MDA-MB-231 breast cancer cells to Taxol either alone or in combination with Dpep and Bpep. The peptides acted with apparent synergism with Taxol on both lines to inhibit growth/survival (MCF7: CI = 0.41 ± 0.04 for Bpep; 0.44 ± 0.04 for Dpep. MDA-MB-231: 0.74 ± 0.03 for Bpep; 0.74 ± 0.05 for Dpep) (Supplementary Figure S5A). Soft agar colony-formation assays with MDA-MB-231 cells showed a similar activity of the combination treatment (Figure 3B). Taxol has been advocated as a potential treatment for glioblastoma [41]. The combination of Bpep and Dpep with Taxol on T98G cells also showed apparent synergy (CI = 0.84 ± 0.06 for Bpep and 0.75 ± 0.07 for Dpep) (Supplementary Figure S5A).

Taxol resistance represents an important clinical challenge [42]. To determine whether the peptides act in this context, we selected Taxol-resistant T98G cells by exposure to increasing concentrations of the drug (Supplementary Figure S5B) and then compared them with non-selected cells for responses to Bpep and Dpep. There was little difference in dose-responses for the peptides in resistant and non-resistant cultures, indicating that Bpep and Dpep were fully effective on Taxol-resistant tumor cells (Supplementary Figure S5B).

The lysosomal inhibitor chloroquine is currently in clinical trials for various cancers [43] and has been reported to enhance the release of cell-penetrating peptides from endosomes [44]. We therefore assessed combinations of chloroquine and Bpep/Dpep in a variety of cancer lines. The results ranged from near-additive (HCT116 cells, CI = 0.92 ± 0.08 for Bpep; 0.87 ± 0.07 for Dpep) to apparent synergy (T98G, CI = 0.56 ± 0.06 for Bpep; 0.75 ± 0.03 for Dpep; MDA-MB-231, CI = 0.59 ± 0.11 for Bpep; 0.47 ± 0.05 for Dpep) (Figure 3C; Supplementary Figure S5C).

The anthracycline doxorubicin is widely used to treat hematopoietic and solid tumors [45]. Combination studies in multiple lines also suggested near-additive effects of combinations of doxorubicin (50 nM) with Bpep and Dpep (Supplementary Figure S6A).

Due to its effects on chromatin structure and cell cycle, doxorubicin has been used to model cell senescence and cell cycle withdrawal [46,47], thus permitting us to ask whether Bpep/Dpep affects survival of non-proliferating cells. Accordingly, multiple lines were pre-treated with or without 100–200 nM doxorubicin for 24 h, then for 6 additional days with various Bpep/Dpep concentrations. Cell counts performed after pretreatment and at the end of study verified that doxorubicin-pretreated MDA-MB-231, A375 and HCT116 cells showed little replication, while T98G and MCF7 cells appeared to slow, but not entirely cease replication (Figure 3D; Supplementary Figure S6B). The anticipated effect of doxorubicin pretreatment on cell accumulation in G2/M was also verified in two lines (HCT116 and T98G) (Supplementary Figure S6C). Comparison of the dose-responses for control and doxorubicin-pretreated cultures revealed them to be essentially identical (Figure 3D; Supplementary Figure S6B). Together, these studies indicate that Bpep/Dpep act additively with doxorubicin to affect tumor cell growth/survival and that they diminish survival of tumor cells irrespective of the proliferative state.

### 3.4. Bpep and Dpep Suppress Expression of Direct CEBPB and CEBPD Targets IL6, IL8 and ASNS

**Figure 4:**
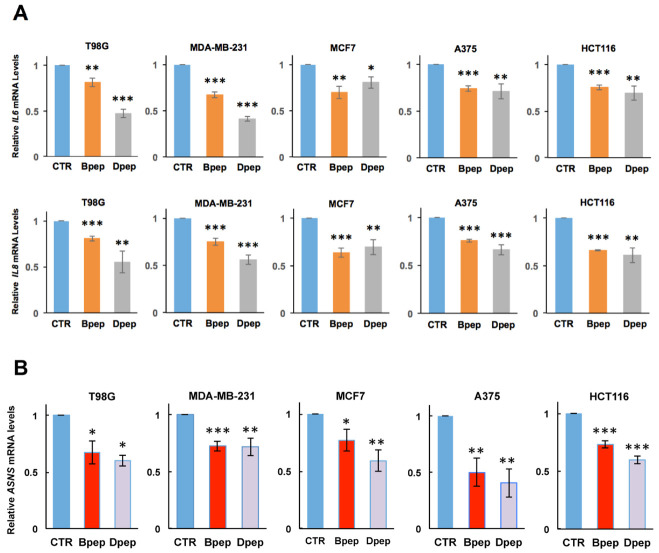
Bpep and Dpep interfered with expression of known CEBPB and CEBPD target genes in multiple cancer lines of various origins. (**A**). Bpep and Dpep suppressed expression of *IL6* and *IL8*. Indicated tumor lines were treated with or without 20 µM Bpep or Dpep for 2 days and then assessed by qPCR for relative levels of *IL6* (upper panel) and *IL8* (lower panel) mRNA. Values represent means ± SEM for 3 experiments, each carried out in triplicate. (**B**). Bpep and Dpep suppressed expression of *ASNS* mRNA. Cultures were treated and assessed as in panel A. *p* values vs. controls are indicated by * as in Figure 1. * *p* < 0.05, ** *p* < 0.005, *** *p* < 0.0005.

If, as anticipated, Bpep and Dpep interfere with CEBPB and CEBPD function, they should affect expression of direct CEBPB/CEBPD targets. *IL6* and *IL8* have been identified as direct CEBPB and CEBPD targets [48,49], and there is evidence that cancer-cell-derived IL-6 and IL-8 promote tumor growth and metastasis and therapeutic resistance [50,51]. We therefore assessed *IL6* and *IL8* transcript levels in multiple cancer lines with or without 48 h of 20 µM Bpep or Dpep exposure. In each case, the peptides significantly reduced *IL6* and *IL8* expression (Figure 4A).

*ASNS*, the gene encoding asparagine synthetase, has been reported as a direct target of ATF5 and CEBPB [52,53], and its over-expression is associated with cancer cell proliferation, metastasis and therapeutic resistance [54]. Assessment of *ASNS* transcripts in the above lines also revealed significant down-regulation after 48 h of 20 µM Bpep or Dpep treatment (Figure 4B). Together, these findings indicate that Bpep/Dpep reduce expression of well-defined ATF5, CEBPB and CEBPD targets with cancer relevance.

### 3.5. Bpep and Dpep Deplete Anti-Apoptotic Protein Survivin and Reduce Expression of Direct ATF5 Targets BCL2 and MCL1

**Figure 5:**
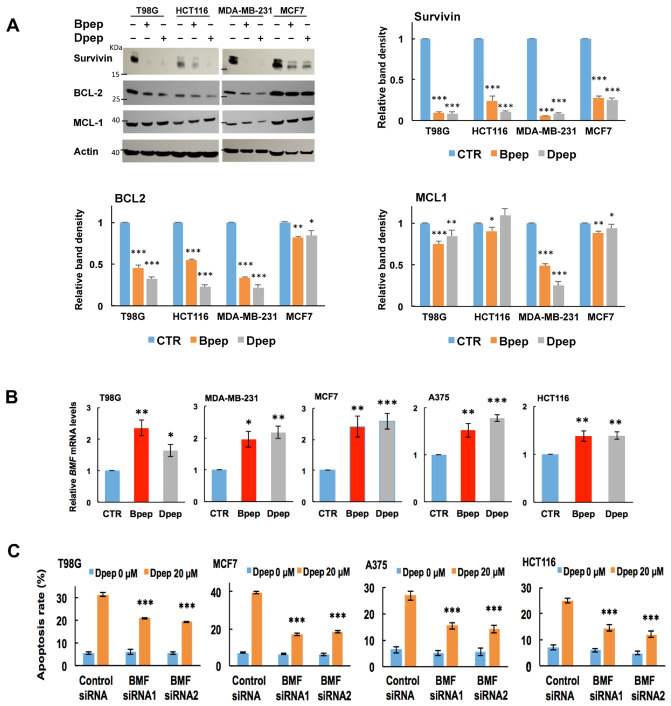
Bpep and Dpep depleted survivin and elevated *BMF* in multiple cancer cell lines of various origins, and BMF was required for Dpep-promoted cancer cell apoptosis. (**A**). Bpep and Dpep suppressed expression of pro-survival proteins, including survivin. Indicated cancer cell lines were exposed to 0 or 20 µM Bpep or Dpep for 3 days and then assessed by Western immunoblotting for levels of BCL2, MCL1 and survivin proteins with actin as a loading control. The upper left panel shows representative blot. Remaining panels show quantification of survivin, BCL2 and MCL1 relative to actin in three independent experiments. *p* values vs. control are indicated by * as in Figure 1. (**B**). Bpep and Dpep upregulated *BMF* in multiple cancer cell lines. Cultures were treated with 0 (CTR) or 20 µM peptides for 2 days as indicated and then assessed by qPCR for relative levels of *BMF* transcripts. Data represent pooled values from 3 independent experiments carried out in triplicate. *p* values vs. control are indicated by * as in Figure 1 (**C**). Knockdown of *BMF* expression with siRNAs protected cancer cell lines from apoptosis promoted by Dpep (20 µM). Data are from one experiment carried out in sextuplicate. Cultures were pretreated with control siRNA, BMF-siRNA1 or BMF-siRNA2 for 2 days and then with 20 µM Dpep for an additional 2 days before assessment for the proportion of cells with apoptotic nuclei. *p* values for peptide-untreated vs. peptide-treated for each condition are indicated by * as in Figure 1. * *p* < 0.05, ** *p* < 0.005, *** *p* < 0.0005.

Survivin (encoded by the *BIRC5* gene) is an anti-apoptotic protein often over-expressed in various cancer types and absent or little-expressed in normal cells [55]. Survivin depletion generally promotes tumor cell apoptotic death, and survivin is therefore recognized as an important therapeutic target [55,56,57]. We reported that dn-ATF5 depletes survivin in multiple tumor lines and that such depletion occurs prior to and is sufficient for their death [58]. We therefore assessed whether Bpep/Dpep would reduce survivin protein expression. Exposure of multiple lines to 20 µM Bpep or Dpep for 72 h promoted profound survivin depletion (Figure 5A).

We additionally examined the effects of the peptides on levels of survival proteins BCL2 and MCL1 in the same lines, which have been described as direct ATF5 targets [6,59]. BCL2 was significantly reduced in all cases (Figure 5A). MCL1 expression also showed significant, but variable, reduction, except in the case of HCT116 cells exposed to Dpep. (Figure 5A). Taken together, these findings identify survivin as a consistent and highly responsive pro-survival target of Bpep and Dpep with additional, more context-dependent effects on ATF5 targets BCL2 and MCL1.

### 3.6. BMF Is Upregulated by Bpep and Dpep and Is Required for Dpep-Promoted Cancer Cell Apoptosis

We next considered whether regulated pro-apoptotic proteins play roles in the apoptotic activities of Bpep/Dpep. A pilot RNAseq study with CP-dn-ATF5-treated T98G cells revealed that among pro-apoptotic genes, encoding *BMF* (BCL2-Modifying Factor) showed by far the largest elevation. To determine whether this was the case for Bpep and Dpep and occurred across multiple tumor cell types, we performed qPCR for *BMF* transcripts in multiple lines exposed to the peptides for 2 days. In all cases, there was a significant elevation of *BMF* expression (Figure 5B). To assess whether BMF plays a required role in Bpep/Dpep activity, we compared apoptosis levels in multiple cultures exposed to the control or two independent BMF siRNAs and then treated with or without Dpep. *BMF* knockdown was approximately 61–86% depending on the cell line (Supplementary Figure S7). In each case, *BMF* knockdown significantly suppressed Dpep-promoted apoptosis by approximately 44–67% (Figure 5C).

### 3.7. Bpep and Dpep Are Active In Vivo

We next assessed whether Bpep/Dpep have efficacy in living animals. Subcutaneous A375 melanoma xenografts were established in SCID mice which were then treated intraperitoneally with Bpep or Dpep (20 mg/kg) 3–4 × /week (Supplementary Figure S8A). There were no evident side effects over the 3-week treatment and no significant differences in weight of treated animals in comparison with vehicle-treated controls. Both peptides significantly reduced growth of the tumors with no major difference between their efficacy.

**Figure 6:**
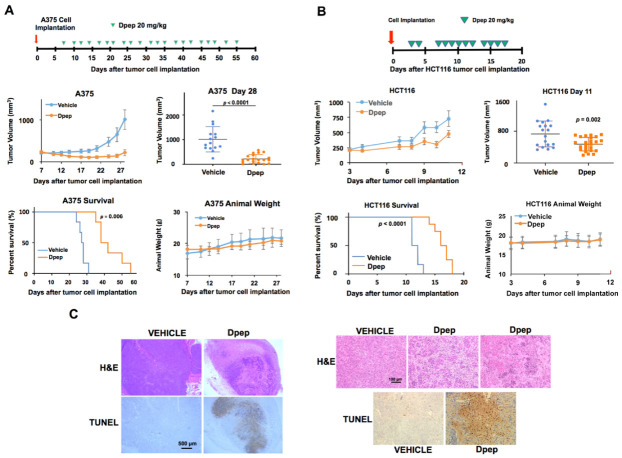
Dpep suppressed tumor growth and prolonged survival in tumor xenograft models. (**A**). Dpep suppressed tumor growth and prolonged survival in the A375 melanoma tumor xenograft model. A375 cells were subcutaneously implanted in the flanks of NCR nude mice. Once tumors formed, the animals were treated intraperitoneally with 20 mg/kg of Dpep or the vehicle, as indicated in the scheme shown in the top panel. Tumor sizes were measured and volumes calculated on each day of injection. The middle left panel shows calculated mean tumor volumes vs. time up to the time (day 28) at which the first vehicle-treated animal reached the experimental endpoint (at least one tumor of calculated volume >1000 mm^3^). The middle right panel compares calculated individual and mean tumor volumes for vehicle and Dpep treated animals on day 28. *p* value vs. vehicle (*n* = 15 tumors): *p* = 1.96 × 10^−5^ for Dpep (*n* = 21 tumors). The lower left panel shows animal survival vs. time for vehicle and Dpep treated animals. Animals were considered to have reached the survival endpoint either when they showed morbid behavior or when at least one tumor reached a calculated volume of >1000 mm^3^. *p* value of 0.0006, determined by a log-rank test. The lower right panel shows mean animal weights vs. time for vehicle and Dpep treated animals to day 28. (**B**). Dpep suppressed tumor growth and prolonged survival in a HCT116 colon cancer xenograft model. HCT116 cells were subcutaneously implanted in the flanks of NCR nude mice. After tumors formed, the animals were treated intraperitoneally with 20 mg/kg of Dpep or vehicle as indicated in the scheme shown in the top panel. The middle left panel shows calculated mean tumor volumes vs. time up to the time (day 11) at which the first vehicle-treated animal reached the experimental endpoint as described in A. The middle right panel compares calculated individual and mean tumor volumes for vehicle and Dpep-treated animals on day 11. *p* value vs. vehicle (*n* = 19 tumors): *p* = 0.002 for Dpep (*n* = 24 tumors). The lower left panel shows animal survival vs. time for vehicle and Dpep-treated animals to day 11. *p* value < 0.0001, determined by log-rank test. The criteria for considering animals to have reached the survival endpoint are as in A. The lower right panel shows mean animal weights vs. time for vehicle and Dpep-treated animals. (**C**). Dpep treatment promoted degeneration and apoptosis of tumor cells in vivo. Subcutaneous A375 xenograft tumors were established in 4 mice as in panel A. When the tumors reached approximately 300 mm^3^, randomly chosen animals were treated with the vehicle or 20 mg/kg of Dpep. The animals were retreated 2 days later, and after an additional 2 days, the animals were sacrificed, and the tumors harvested for H&E and TUNEL staining. The left panels show a low-power view of staining of nearby sections from vehicle-treated animal A, tumor 1 and Dpep-treated animal D, tumor 2. The upper right panels show a higher power view of H&E stained sections from vehicle-treated animal A, tumor 1; Dpep-treated animal C, tumor 1 (center) and Dpep-treated animal D, tumor 2. The lower right panels show TUNEL-stained sections from vehicle-treated animal A, tumor 1 and Dpep-treated animal C, tumor 1.

Because of the similar actions of Bpep and Dpep, subsequent experiments employed Dpep. To determine whether elevating Dpep dosing might increase anti-tumor activity, we compared effects of 20 and 50 mg/kg in mice bearing subcutaneously implanted B16-F10 murine melanoma cell tumors (Supplementary Figure S8B**).** While both doses significantly slowed tumor growth, there was no significant difference in their efficacy. There were also no evident side effects or differences in animal weight compared with controls over the 9-day treatment regimen for either dose. Next, using the A375 melanoma xenograft model, we asked whether Dpep (20 mg/kg) would affect survival in addition to tumor growth over a more extended treatment period (Figure 6A). As in our initial study, Dpep suppressed tumor growth up to 3 weeks of treatment (the time point when mice in the control group first reached the survival endpoint). With continued peptide dosing, there was a highly significant prolongation of survival time compared with controls. To assess efficacy in a second animal model, we employed subcutaneously implanted HCT116 cell xenografts (Figure 6B). Here also, despite more aggressive tumor growth, Dpep (20 mg/kg) significantly decreased tumor growth rate and prolonged survival time.

To verify that Dpep affects tumor cell survival in vivo as in vitro, we examined A375 xenograft tumors after two treatments with the peptide. H&E staining revealed widespread cell degeneration, while TUNEL staining indicated extensive apoptotic death compared with tumors from vehicle-treated animals (Figure 6C; Supplementary Figure S8C).

In addition, to monitor possible off-target actions of Dpep, we examined H&E-stained tissues from multiple organs (heart, lung, liver, kidney, spleen, brain, pancreas and intestine) of animals at the endpoint of the study shown in Figure 6A after a full course of treatment with the peptide or vehicle. In all cases, there were no discernable differences between the tissues from peptide- and vehicle-treated animals (see Supplementary Figure S9). Examination of heart, lung and liver sections from animals in the study described in Figure 6B also showed no differences between vehicle- and peptide-treated animals (data not shown).

## 4. Discussion

Numerous studies have associated ATF5, CEBPB and CEBPD with various aspects of malignancy, including patient prognosis and survival [1,2,3,4,5,6,7,8,9,10,11,12,13,14,15,16,17,18,19,20,21,22,23,24,25,26,27,28,29,30,31,32,33]. To target these transcription factors in a potentially clinically relevant way, we designed cell-penetrating dn peptides (Bpep and Dpep) to act as function-blocking decoys. The peptides promote apoptotic death of a wide range of tumor types, but not non-transformed cells; work additively or synergistically combined with other therapeutics; and, in animal models, inhibit tumor growth and increase survival without apparent side effects.

In past work, we characterized anti-tumor activity of a cell-penetrating dn-ATF5 [31,32]. A relevant question is why Bpep and Dpep may differ from CP-dn-ATF5 with respect to potential therapeutic value and merit further development. Firstly, as noted above, dn-ATF5 interacts with CEBPB and CEBPD in a leucine-zipper-dependent manner [33]. This strongly supports the idea that Bpep and Dpep conversely associate with ATF5. In contrast, dn-ATF5 does not associate with endogenous ATF5 [33]. Thus, while dn-ATF5 inhibits CEBPB and CEBPD function, Bpep and Dpep should inhibit not only CEBPB and CEBPD function, but also that of ATF5. This distinction is potentially important. ATF5 has been experimentally linked to cancer cell growth and survival, and its expression is associated with outcome in at least several cancers [2,3,4,5,6,12,13,15,18,22]. Furthermore, a novel DNA binding site for ATF5 has been identified [60], suggesting it regulates genes distinct from those that CEBPB and CEBPD regulate. In addition, ATF5 is characterized as an important player in the mitochondrial stress response, of relevance to tumor cell survival [61]. Moreover, in a non-transcriptional role, ATF5 has been reported as a critical component of the centrosome [62], also of relevance to cancer cell survival/growth. We are not aware of such roles for CEBPB or CEBPD. In addition, other cancer-relevant proteins are potentially bound and sequestered by Bpep and Dpep, but not by dn-ATF5. As an example, CEBPB forms functional heterodimers with ATF4, another transcription factor that protects cancer cells under stress conditions [63]. Further studies are warranted to better define the target differences between Dpep, Bpep and dn-ATF5. Finally, of importance, Dpep and Bpep show 5–10-fold greater potency compared with CP-dn-ATF5 [32].

Key features of Bpep and Dpep are the possession of the CEBPB and CEBPD leucine zipper sequence, respectively. Replacement of the heptad repeat leucines in these sequences eliminated or severely decreased anti-tumor cell activity, supporting the idea that Bpep/Dpep function via specific zipper–zipper interactions. Such dependence also reinforces the absence of non-specific toxicity by the peptides.

Although not identical, Bpep and Dpep show quite comparable activities. This includes similar efficacy and potency across multiple cell lines and in combination treatments, additive effects when tested in combination with one another and comparable in vivo efficacy. These findings suggest similar mechanisms of action. However, given that the peptides did not act identically in some situations, one cannot not rule out the possibility that there may be conditions or cancer types in which one peptide is significantly more effective than the other.

Bpep and Dpep show efficacy as monotherapies and in combinations with other cancer treatments. In each combination tested, the peptides did not interfere with partner activity and acted additively or synergistically. If, as appears, the peptides have little toxicity on their own, this supports the idea that they can be combined with therapeutics that have limiting side effects in order to achieve efficacy with partner doses below those causing toxicity.

An important concept in cancer therapeutics is that tumor cells may temporarily withdraw from the cell cycle, potentially for extended times, and thereby escape treatments that target rapidly dividing cells to seed metastasis and recurrence [64,65]. Here, we found that Bpep/Dpep inhibited survival of cancer cells rendered slowly- or non-replicating by pre-treatment with doxorubicin. This indicates that the peptides can cause death of tumor cells that are not actively in the cell cycle. Further studies will be needed to more fully define the range of tumor cell replication states that are susceptible to the peptides.

We found that Bpep and Dpep significantly reduced expression of known direct target genes of CEBPB (*IL6, IL8, ASNS*), CEBPD (*IL6, IL8*) and ATF5 (*ASNS*) as well as of proteins (BCL2 and MCL1) encoded by ATF5 target genes. While further studies will be required to verify that the peptides reduce association of the three transcription factors with their target genes, these findings support the idea that Bpep and Dpep interfere with the expression of genes regulated by CEBPB, CEBPD and ATF5. Beyond representing markers for Bpep and Dpep activity, IL6, IL8 and ASNS have potential relevance for cancer treatment. IL-6 and IL-8, derived either from tumors or their niche (which should also take up Bpep/Dpep), contribute to tumor growth, metastasis and therapeutic resistance [50,51]. ASNS over-expression is associated with aggressive tumor growth, while ASNS knockdown inhibits growth in certain tumor models [54]. Moreover, ATF5 gene polymorphisms that appear to enhance activity at the ASNS promoter are linked to asparaginase treatment resistance in childhood acute lymphoblastic leukemia [8]. These findings represent additional mechanisms that may contribute to the efficacy of Bpep and Dpep for cancer treatment, both by direct actions on tumor cells and their environment.

While we have yet to define the full set of molecular mechanisms by which Bpep and Dpep selectively kill tumor cells, our findings shed light on distal events leading to apoptosis. One is profound depletion of survivin, a member of the Inhibitor of Apoptosis (IAP) family with additional roles in cell proliferation and mitochondrial stress [55]. Survivin down-regulation is sufficient to interfere with cancer cell growth and survival in vitro and in vivo, and survivin is recognized as an attractive target for cancer treatment [55,56,57]. Another significant response is up-regulation of *BMF*, a pro-apoptotic member of the BCL2 family. Though BMF has received relatively less attention than other pro-apoptotic proteins in the context of cancer, an increasing amount of literature links its regulation to mechanisms of anti-cancer agents [66]. In addition to observing that the peptides significantly elevate *BMF* in multiple tumor lines, we found that *BMF* knockdown was sufficient to suppress their apoptotic activities. These findings thus indicate that Bpep and Dpep trigger apoptosis by setting in motion a set of molecular events that lead to survivin depletion and BMF activation.

The aim of this study was to evaluate the potentials of Bpep and Dpep as novel prototypic drugs for cancer treatment. Our in vitro studies verified their selective efficacy on a range of tumor cell types. We further observed efficacy in suppressing tumor growth in 3 different in vivo models, and in two survival studies, there was highly significant prolongation of survival time. We found that doses of 20 and 50 mg/kg had similar efficacy in suppressing tumor growth. It remains to be determined whether lower doses or a different treatment regimen would be equally or more effective. Even with prolonged treatments lasting more than 6 weeks, we did not observe loss of weight or other evident side effects in the animal subjects. Although more detailed studies will be required to fully examine in vivo safety of the peptides, the current findings point to a high degree of tolerability.

## 5. Conclusions

On the basis of the work described here, we conclude that the novel cell-penetrating peptides Bpep and Dpep promote apoptotic death of a wide range of cancer cells, both in vitro and in vivo without such effects on non-transformed cells. The distal part of the mechanism by which they do so includes depletion of the survival protein survivin and elevation of death protein BMF. Our findings support the idea that Bpep and Dpep or modified forms thereof have potential for treatment of a variety of cancer types. Our data also support their use in combination therapies in which they can augment the actions of other anti-cancer agents and/or reduce the levels needed to achieve efficacy.

## Data Availability

The data presented in this study are available on request from the corresponding author.

## Supplementary Materials

The following are available online at https://www.mdpi.com/article/10.3390/cancers13102504/s1, Figure S1: Bpep and Dpep activities on cultured tumor cells; Figure S2: Images of Bpep and Dpep activities on tumor and non-transformed cells; Figure S3: Effects of Bpep and Dpep on colony formation, apoptosis and migration of cultured tumor cells; Figure S4: Bpep and Dpep act synergistically with gamma radiation; Figure S5: Effects of Dpep and Bpep in combinations with Taxol and chloroquine and on Taxol-resistant cells; Figure S6: Effects of Bpep and Dpep on cancer cells in combination with doxorubicin and on doxorubicin pre-treated cells; Figure S7: Knockdown of *BMF* by BMF-siRNA1 and BMF-siRNA2 in multiple cancer cell lines; Figure S8: Bpep and Dpep inhibit growth and survival of melanoma cells in vivo; Figure S9: Dpep treatment does not affect the histological features of normal tissues.

## References

[1] Gomis R.R., Alarcón C., Nadal C., Van Poznak C., Massagué J. (2006). C/EBPβ at the core of the TGFβ cytostatic response and its evasion in metastatic breast cancer cells. Cancer Cell.

[2] Angelastro J.M., Canoll P.D., Kuo J., Weicker M., Costa A., Bruce J.N., A Greene L. (2005). Selective destruction of glioblastoma cells by interference with the activity or expression of ATF5. Oncogene.

[3] Piva R., Pellegrino E., Mattioli M., Agnelli L., Lombardi L., Boccalatte F., Costa G., Ruggeri B.A., Cheng M., Chiarle R. (2006). Functional validation of the anaplastic lymphoma kinase signature identifies CEBPB and Bcl2A1 as critical target genes. J. Clin. Investig..

[4] Monaco S.E., Angelastro J.M., Szabolcs M., Greene L.A. (2007). The transcription factor ATF5 is widely expressed in carcinomas, and interference with its function selectively kills neoplastic, but not nontransformed, breast cell lines. Int. J. Cancer.

[5] Oh W.J., Rishi V., Orosz A., Gerdes M.J., Vinson C. (2007). Inhibition of CCAAT/Enhancer Binding Protein Family DNA Binding in Mouse Epidermis Prevents and Regresses Papillomas. Cancer Res..

[6] Sheng Z., Li L., Zhu L.J., Smith T.W., Demers A., Ross A.H., Moser R.P., Green M.R. (2010). A genome-wide RNA interference screen reveals an essential CREB3L2-ATF5-MCL1 survival pathway in malignant glioma with therapeutic implications. Nat. Med..

[7] Carro M.S., Lim W.K., Alvarez M.J., Bollo R.J., Zhao X., Snyder E.Y., Sulman E.P., Anne S.L., Doetsch F., Colman H. (2009). The transcriptional network for mesenchymal transformation of brain tumours. Nature.

[8] Rousseau J., Gagné V., Labuda M., Beaubois C., Sinnett D., Laverdière C., Moghrabi A., Sallan S.E., Silverman L.B., Neuberg D. (2011). ATF5 polymorphisms influence ATF function and response to treatment in children with childhood acute lymphoblastic leukemia. Blood.

[9] Sarkar T.R., Sharan S., Wang J., Pawar S.A., Cantwell C.A., Johnson P.F., Morrison D.K., Wang J.-M., Sterneck E. (2011). Identification of a Src Tyrosine Kinase/SIAH2 E3 Ubiquitin Ligase Pathway That Regulates C/EBP Expression and Contributes to Transformation of Breast Tumor Cells. Mol. Cell. Biol..

[10] Hu M., Wang B., Qian D., Li L., Zhang L., Song X., Liu D.X. (2012). Interference with ATF5 function enhances the sensitivity of human pancreatic cancer cells to paclitaxel-induced apoptosis. Anticancer Res..

[11] Wang Y.-H., Wu W.-J., Wang W.-J., Huang H.-Y., Li W.-M., Yeh B.-W., Wu T.-F., Shiue Y.-L., Sheu J.J.-C., Wang J.-M. (2015). CEBPDamplification and overexpression in urothelial carcinoma: A driver of tumor metastasis indicating adverse prognosis. Oncotarget.

[12] Ishihara S., Yasuda M., Ishizu A., Ishikawa M., Shirato H., Haga H. (2015). Activating transcription factor 5 enhances radioresistance and malignancy in cancer cells. Oncotarget.

[13] Nukuda A., Endoh H., Yasuda M., Mizutani T., Kawabata K., Haga H. (2016). Role of ATF5 in the invasive potential of diverse human cancer cell lines. Biochem. Biophys. Res. Commun..

[14] Banerjee S., Aykin-Burns N., Krager K.J., Shah S.K., Melnyk S.B., Hauer-Jensen M., Pawar S.A. (2016). Loss of C/EBPδ enhances IR-induced cell death by promoting oxidative stress and mitochondrial dysfunction. Free Radic. Biol. Med..

[15] Angelastro J.M. (2017). Targeting ATF5 in Cancer. Trends Cancer.

[16] Gardiner J.D., Abegglen L.M., Huang X., Carter B.E., Schackmann E.A., Stucki M., Paxton C.N., Randall R.L., Amatruda J.F., Putnam A.R. (2017). C/EBPβ-1 promotes transformation and chemoresistance in Ewing sarcoma cells. Oncotarget.

[17] Zhang Y., Wang H.-R., Wrana J.L. (2004). Smurf1: A Link between Cell Polarity and Ubiquitination. Cell Cycle.

[18] Ben-Shmuel S., Rashed R., Rostoker R., Isakov E., Shen-Orr Z., Leroith D. (2017). Activating Transcription Factor-5 Knockdown Reduces Aggressiveness of Mammary Tumor Cells and Attenuates Mammary Tumor Growth. Front. Endocrinol..

[19] Wang W.-J., Li C.-F., Chu Y.-Y., Wang Y.-H., Hour T.-C., Yen C.-J., Chang W.-C., Wang J.-M. (2017). Inhibition of the EGFR/STAT3/CEBPD Axis Reverses Cisplatin Cross-resistance with Paclitaxel in the Urothelial Carcinoma of the Urinary Bladder. Clin. Cancer Res..

[20] Messenger Z.J., Hall J.R., Jima D.D., House J.S., Tam H.W., Tokarz D.A., Smart R.C. (2018). C/EBPβ deletion in oncogenic Ras skin tumors is a synthetic lethal event. Cell Death Dis..

[21] Liu D., Zhang X.-X., Li M.-C., Cao C.-H., Wan D.-Y., Xi B.-X., Tan J.-H., Wang J., Yang Z.-Y., Feng X.-X. (2018). C/EBPβ enhances platinum resistance of ovarian cancer cells by reprogramming H3K79 methylation. Nat. Commun..

[22] Feldheim J., Kessler A.F., Schmitt D., Wilczek L., Linsenmann T., Dahlmann M., Monoranu C.M., Ernestus R.-I., Hagemann C., Löhr M. (2018). Expression of activating transcription factor 5 (ATF5) is increased in astrocytomas of different WHO grades and correlates with survival of glioblastoma patients. OncoTargets Ther..

[23] Wang F., Gao Y., Tang L., Ning K., Geng N., Zhang H., Li Y., Li Y., Liu F., Li F. (2019). A novel PAK4-CEBPB-CLDN4 axis involving in breast cancer cell migration and invasion. Biochem. Biophys. Res. Commun..

[24] Du Q., Tan Z., Shi F., Tang M., Xie L., Zhao L., Li Y., Hu J., Zhou M., Bode A. (2019). PGC1α/CEBPB/CPT1A axis promotes radiation resistance of nasopharyngeal carcinoma through activating fatty acid oxidation. Cancer Sci..

[25] Wang D., Cheng X., Li Y., Guo M., Zhao W., Qiu J., Zheng Y., Meng M., Ping X., Chen X. (2019). C/EBPδ-Slug-Lox1 axis promotes metastasis of lung adenocarcinoma via oxLDL uptake. Oncogene.

[26] Wang D., Ruan X., Liu X., Xue Y., Shao L., Yang C., Zhu L., Yang Y., Li Z., Yu B. (2020). SUMOylation of PUM2 promotes the vasculogenic mimicry of glioma cells via regulating CEBPD. Clin. Transl. Med..

[27] Wu H., Gu J., Zhou D., Cheng W., Wang Y., Wang Q., Wang X. (2020). LINC00160 mediated paclitaxel-And doxorubicin-resistance in breast cancer cells by regulating TFF3 via transcription factor C/EBPβ. J. Cell. Mol. Med..

[28] Kudo T., Prentzell M.T., Mohapatra S.R., Sahm F., Zhao Z., Grummt I., Wick W., Opitz C.A., Platten M., Green E.W. (2020). Constitutive Expression of the Immunosuppressive Tryptophan Dioxygenase TDO2 in Glioblastoma Is Driven by the Transcription Factor C/EBPβ. Front. Immunol..

[29] Hua Z.-Y., Hansen J.N., He M., Dai S.-K., Choi Y., Fulton M.D., Lloyd S.M., Szemes M., Sen J., Ding H.-F. (2020). PRMT1 promotes neuroblastoma cell survival through ATF5. Oncogenesis.

[30] Arias A., Lamé M.W., Santarelli L., Hen R., A Greene L., Angelastro J.M. (2011). Regulated ATF5 loss-of-function in adult mice blocks formation and causes regression/eradication of gliomas. Oncogene.

[31] Cates C.C., Arias A.D., Wong L.S.N., Lamé M.W., Sidorov M., Cayanan G., Rowland D.J., Fung J., Karpel-Massler G., Siegelin M.D. (2016). Regression/Eradication of gliomas in mice by a systemically-deliverable ATF5 dominant-negative peptide. Oncotarget.

[32] Karpel-Massler G., Horst B.A., Shu C., Chau L., Tsujiuchi T., Bruce J.N., Canoll P., Greene L.A., Angelastro J.M., Siegelin M.D. (2016). A Synthetic Cell-Penetrating Dominant-Negative ATF5 Peptide Exerts Anticancer Activity against a Broad Spectrum of Treatment-Resistant Cancers. Clin. Cancer Res..

[33] Sun X., Jefferson P., Zhou Q., Angelastro J.M., Greene L.A. (2020). Dominant-Negative ATF5 Compromises Cancer Cell Survival by Targeting CEBPB and CEBPD. Mol. Cancer Res..

[34] Olive M., Williams S.C., Dezan C., Johnson P.F., Vinson C. (1996). Design of a C/EBP-specific, Dominant-negative bZIP Protein with Both Inhibitory and Gain-of-function Properties. J. Biol. Chem..

[35] Reinke A.W., Baek J., Ashenberg O., Keating A.E. (2013). Networks of bZIP Protein-Protein Interactions Diversified Over a Billion Years of Evolution. Science.

[36] Vinson C.R., Hai T., Boyd S.M. (1993). Dimerization specificity of the leucine zipper-containing bZIP motif on DNA binding: Prediction and rational design. Genes Dev..

[37] Dupont E., Prochiantz A., Joliot A. (2015). Penetratin Story: An Overview. Methods Mol. Biol..

[38] Sonabend A.M., Yun J., Lei L., Leung R., Soderquist C., Crisman C., Gill B.J., Carminucci A., Sisti J., Castelli M. (2013). Murine cell line model of proneural glioma for evaluation of anti-tumor therapies. J. Neurooncol..

[39] Beaulieu J.-F., Ménard D. (2012). Isolation, Characterization, and Culture of Normal Human Intestinal Crypt and Villus Cells. Methods Mol. Biol..

[40] Zhou Q., Chai W. (2016). Suppression of STN1 enhances the cytotoxicity of chemotherapeutic agents in cancer cells by elevating DNA damage. Oncol. Lett..

[41] Ullah I., Chung K., Bae S., Li Y., Kim C., Choi B., Nam H.Y., Kim S.H., Yun C.-O., Lee K.Y. (2020). Nose-to-Brain Delivery of Cancer-Targeting Paclitaxel-Loaded Nanoparticles Potentiates Antitumor Effects in Malignant Glioblastoma. Mol. Pharm..

[42] Rodrigues-Ferreira S., Moindjie H., Haykal M.M., Nahmias C. (2020). Predicting and Overcoming Taxane Chemoresistance. Trends Mol. Med..

[43] Xu R., Ji Z., Xu C., Zhu J. (2018). The clinical value of using chloroquine or hydroxychloroquine as autophagy inhibitors in the treatment of cancers. Medicine.

[44] Caron N.J., Quenneville S.P., Tremblay J.P. (2004). Endosome disruption enhances the functional nuclear delivery of Tat-fusion proteins. Biochem. Biophys. Res. Commun..

[45] Van Der Zanden S.Y., Qiao X., Neefjes J. (2020). New insights into the activities and toxicities of the old anticancer drug doxorubicin. FEBS J..

[46] A Fornari F., Jarvis W.D., Grant S., Orr M.S., Randolph J.K., White F.K., Mumaw V.R., Lovings E.T., Freeman R.H., A Gewirtz D. (1994). Induction of differentiation and growth arrest associated with nascent (nonoligosomal) DNA fragmentation and reduced c-myc expression in MCF-7 human breast tumor cells after continuous exposure to a sublethal concentration of doxorubicin. Cell Growth Differ..

[47] Bojko A., Czarnecka-Herok J., Charzynska A., Dabrowski M., Sikora E. (2019). Diversity of the Senescence Phenotype of Cancer Cells Treated with Chemotherapeutic Agents. Cells.

[48] Kuilman T., Michaloglou C., Vredeveld L.C., Douma S., Van Doorn R., Desmet C.J., Aarden L.A., Mooi W.J., Peeper D.S. (2008). Oncogene-Induced Senescence Relayed by an Interleukin-Dependent Inflammatory Network. Cell.

[49] Hungness E.S., Luo G.-J., Pritts T.A., Sun X., Robb B.W., Hershko D., Hasselgren P.-O. (2002). Transcription factors C/EBP-beta and -delta regulate IL-6 production in IL-1beta-stimulated human enterocytes. J. Cell. Physiol..

[50] Taher M.Y., Davies D.M., Maher J. (2018). The role of the interleukin (IL)-6/IL-6 receptor axis in cancer. Biochem. Soc. Trans..

[51] David J., Dominguez C., Hamilton D.H., Palena C. (2016). The IL-8/IL-8R Axis: A Double Agent in Tumor Immune Resistance. Vaccines.

[52] Al Sarraj J., Vinson C., Thiel G. (2005). Regulation of asparagine synthetase gene transcription by the basic region leucine zipper transcription factors ATF5 and CHOP. Biol. Chem..

[53] Siu F., Chen C., Zhong C., Kilberg M.S. (2001). CCAAT/Enhancer-binding Protein-β Is a Mediator of the Nutrient-sensing Response Pathway That Activates the Human Asparagine Synthetase Gene. J. Biol. Chem..

[54] Chiu M., Taurino G., Bianchi M.G., Kilberg M.S., Bussolati O. (2020). Asparagine Synthetase in Cancer: Beyond Acute Lymphoblastic Leukemia. Front. Oncol..

[55] Wheatley S.P., Altieri D.C. (2019). Survivin at a glance. J. Cell Sci..

[56] Warrier N.M., Agarwal P., Kumar P. (2020). Emerging Importance of Survivin in Stem Cells and Cancer: The Development of New Cancer Therapeutics. Stem Cell Rev. Rep..

[57] García D.M., Manero-Rupérez N., Quesada R., Korrodi-Gregório L., Soto-Cerrato V. (2019). Therapeutic strategies involving survivin inhibition in cancer. Med. Res. Rev..

[58] Sun X., Angelastro J.M., Merino D., Zhou Q., Siegelin M.D., Greene L.A. (2019). Dominant-negative ATF5 rapidly depletes survivin in tumor cells. Cell Death Dis..

[59] Dluzen D., Li G., Tacelosky D., Moreau M., Liu D.X. (2011). BCL-2 Is a Downstream Target of ATF5 That Mediates the Prosurvival Function of ATF5 in a Cell Type-dependent Manner. J. Biol. Chem..

[60] Li G., Li W., Angelastro J.M., Greene L.A., Liu D.X. (2009). Identification of a Novel DNA Binding Site and a Transcriptional Target for Activating Transcription Factor 5 in C6 Glioma and MCF-7 Breast Cancer Cells. Mol. Cancer Res..

[61] Fiorese C., Schulz A.M., Lin Y.-F., Rosin N., Pellegrino M.W., Haynes C.M. (2016). The Transcription Factor ATF5 Mediates a Mammalian Mitochondrial UPR. Curr. Biol..

[62] Madarampalli B., Yuan Y., Liu D., Lengel K., Xu Y., Li G., Yang J., Liu X., Lu Z., Liu D.X. (2015). ATF5 Connects the Pericentriolar Materials to the Proximal End of the Mother Centriole. Cell.

[63] Mann I.K., Chatterjee R., Zhao J., He X., Weirauch M.T., Hughes T.R., Vinson C. (2013). CG methylated microarrays identify a novel methylated sequence bound by the CEBPB ATF4 heterodimer that is active in vivo. Genome Res..

[64] Summers M.A., McDonald M.M., Croucher P.I. (2020). Cancer Cell Dormancy in Metastasis. Cold Spring Harb. Perspect. Med..

[65] Park S.-Y., Nam J.-S. (2020). The force awakens: Metastatic dormant cancer cells. Exp. Mol. Med..

[66] Kurppa K.J., Liu Y., To C., Zhang T., Fan M., Vajdi A., Knelson E.H., Xie Y., Lim K., Cejas P. (2020). Treatment-Induced Tumor Dormancy through YAP-Mediated Transcriptional Reprogramming of the Apoptotic Pathway. Cancer Cell.

