# Supplementary material for: Cell-Penetrating CEBPB and CEBPD Leucine Zipper Decoys as Broadly Acting Anti-Cancer Agents
Source: Cancers (Basel). 2021 May 20;13(10):2504. doi: 10.3390/cancers13102504 (PMC8161188; doi:10.3390/cancers13102504)
Supplement: Supplementary file 1 [file cancers-13-02504-s001.zip › cancers-1202275-supplementary 2/Supplementary Figure S2+legend 5-21.pdf]

Supplementary Fig S2

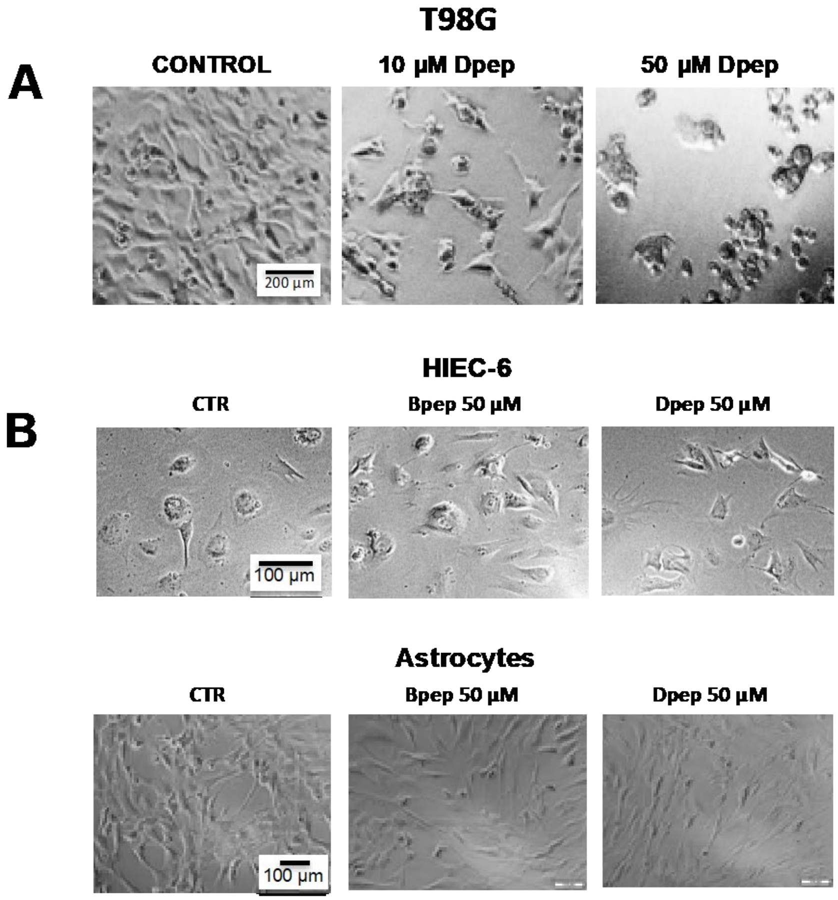

Supplementary Figure S2. **Images of Bpep and Dpep activities on tumor and non-transformed cells**. **(A)**. Images of T98G cell cultures treated for 6 days with 0, 10 or 50 µM concentrations of Dpep. **(B).** Images of non-transformed HIEC-6 and human astrocytes after 6 days of exposure to 50 µM Bpep or Dpep.
