# Supplementary material for: Cell-Penetrating CEBPB and CEBPD Leucine Zipper Decoys as Broadly Acting Anti-Cancer Agents
Source: Cancers (Basel). 2021 May 20;13(10):2504. doi: 10.3390/cancers13102504 (PMC8161188; doi:10.3390/cancers13102504)
Supplement: Supplementary file 1 [file cancers-13-02504-s001.zip › cancers-1202275-supplementary 2/Supplementary Figure S4 + legend 5-21.pdf]

Supplementary Fig S4

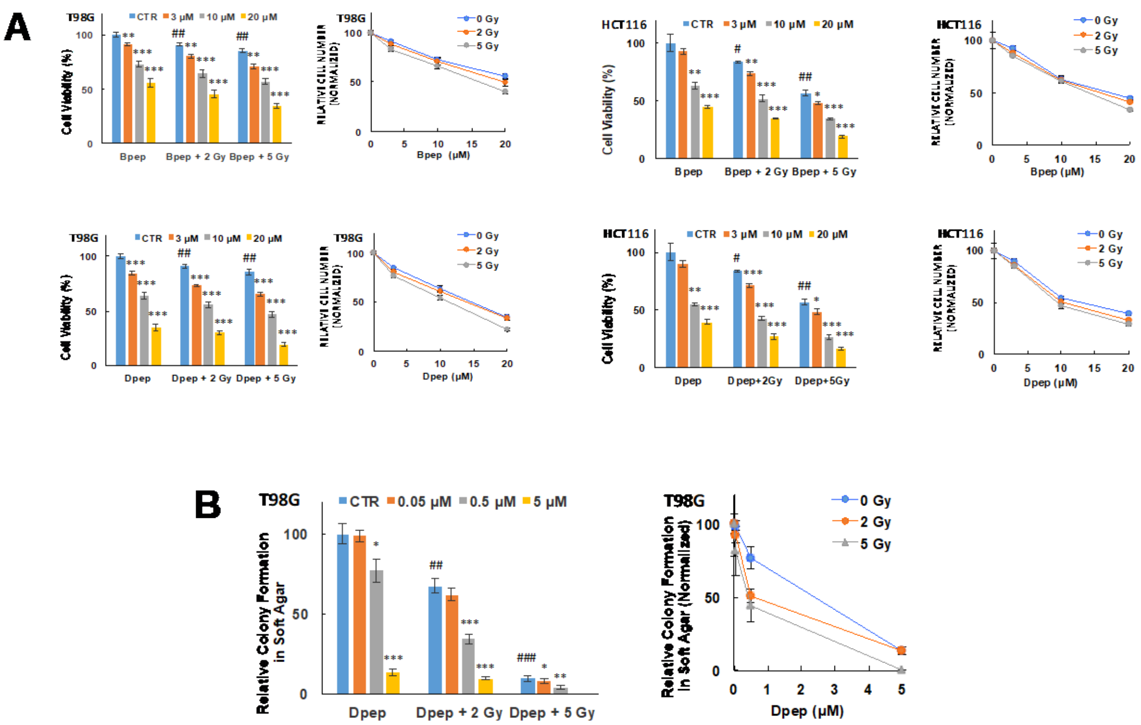

Supplementary Figure S4. **Bpep and Dpep act synergistically with gamma radiation**. **(A).** Bpep and Dpep act additively to synergistically on cancer cells pre-treated with gamma radiation. Replicate cultures of T98G and HCT116 cells were exposed to the indicated levels of radiation. One day later, the cultures were treated with the indicated concentrations of Bpep and Dpep, maintained for 6 days, and then assessed for cell number. Data are from one experiment carried out in triplicate. Bar graphs show relative cell numbers for each condition. Line graphs show normalized response as described in legend to Figure 3A. p values are as described in Figures 1 and 3. **(B).** Dpep acts additively to synergistically in combination with gamma radiation to inhibit colony formation by T98G cells on soft agar. Replicate cultures were treated in combination with the indicated concentrations of Dpep and gamma radiation and assessed 12 days later for relative colony formation. Data are from one experiment carried out in triplicate. Bar graph shows relative colony number for each condition. Line graph shows normalized response as described in legend to Figure 3A. p values are as described above.
