# Supplementary material for: Cell-Penetrating CEBPB and CEBPD Leucine Zipper Decoys as Broadly Acting Anti-Cancer Agents
Source: Cancers (Basel). 2021 May 20;13(10):2504. doi: 10.3390/cancers13102504 (PMC8161188; doi:10.3390/cancers13102504)
Supplement: Supplementary file 1 [file cancers-13-02504-s001.zip › cancers-1202275-supplementary 2/Supplementary Figure S5 + legend 5-21.pdf]

Supplementary Fig S5

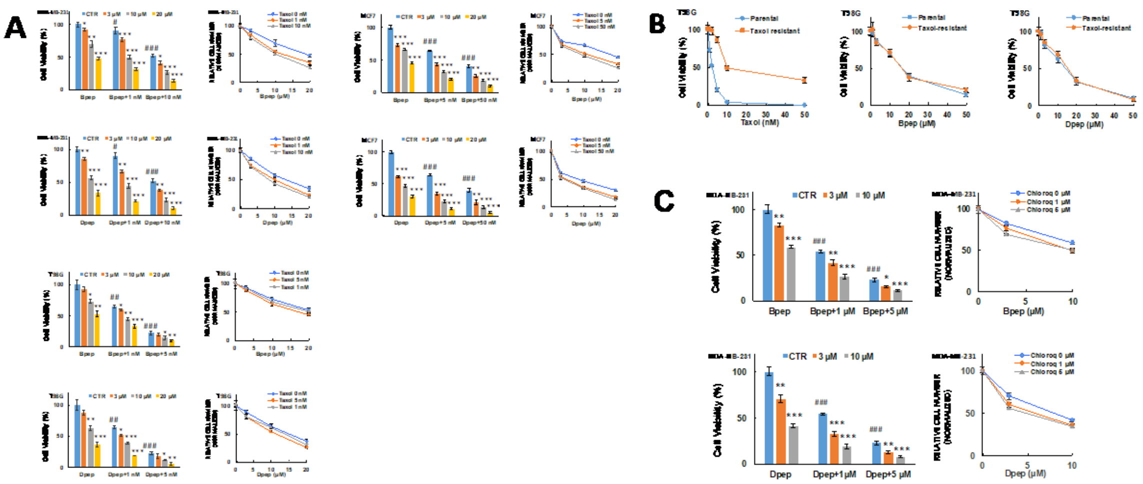

Supplementary Figure S5. **Effects of Dpep and Bpep in combinations with Taxol and chloroquine and on Taxol-resistant cells**. **(A)**. Bpep and Dpep act synergistically to additively in combination with Taxol on multiple cancer cell lines. Replicate cultures were treated in combination with indicated concentrations of Bpep or Dpep and Taxol and assessed 6 days later for relative cell number. Data are from one of two independent experiments, each in triplicate, with comparable results. Bar graphs show relative cell numbers for each condition; line graphs show normalized response as described in legend to Figure 3A. p values are indicated as described in Figures 1 and 3. **(B).** Bpep and Dpep show comparable potency in suppression of growth/survival of wild-type and taxol-resistance T98G cells. Data are from one of two independent experiments, each in triplicate, with comparable results. Left panel compares Taxol sensitivity of wild-type and taxol-resistance T98G cells. Center and right panels show dose-response relationships for responses of wild type and Taxol-resistant T98G cells to Bpep and Dpep, respectively. **(C).** Bpep and D pep appear to work synergistically with chloroquine in cultures of MDA-MB-231 cells. Data are from one of two independent experiments, each in triplicate, with comparable results. Replicate cultures were treated for 6 days in combination with the indicated concentrations of chloroquine and and Bpep or Dpep and assessed for relative cell numbers. Bar graphs show relative cell numbers for each condition; line graphs show normalized response as described in legend to Figure 3A. p values vs control are indicated as described in Figures 1 and 3
