# Supplementary material for: Cell-Penetrating CEBPB and CEBPD Leucine Zipper Decoys as Broadly Acting Anti-Cancer Agents
Source: Cancers (Basel). 2021 May 20;13(10):2504. doi: 10.3390/cancers13102504 (PMC8161188; doi:10.3390/cancers13102504)
Supplement: Supplementary file 1 [file cancers-13-02504-s001.zip › cancers-1202275-supplementary 2/Supplementary Figure S6. + legend 5-21.pdf]

Supplementary Fig S6

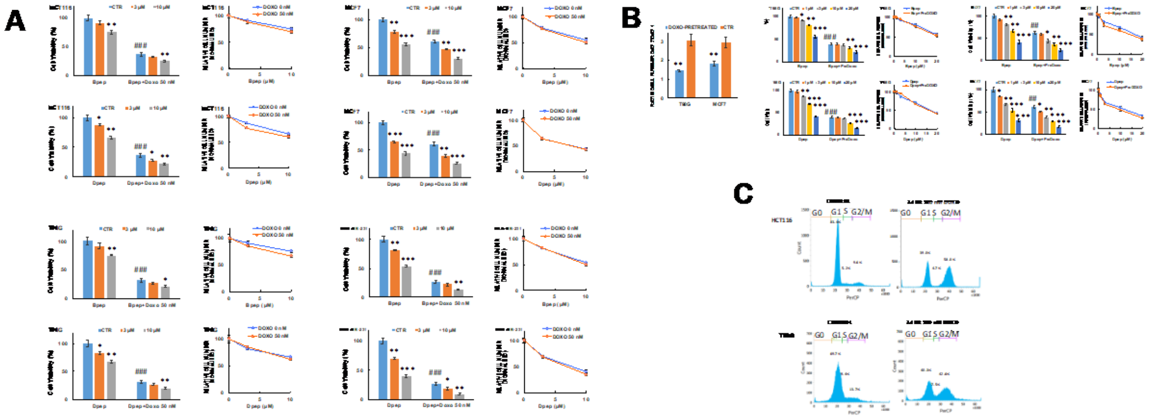

Supplementary Figure S6. **Effects of Bpep and Dpep on cancer cells in combination with doxorubicin and on doxorubicin pre-treated cells**. (A). Bpep and Dpep work additively to inhibit growth/survival of multiple cancer cell lines in combination with doxorubicin. Replicate cultures were treated for 6 days with 0, 3, or 10 μM Bpep or Dpep in combination with 0 or 50 nM doxorubicin and assessed for relative cell numbers. Data are from one experiment carried out in triplicate. Bar graphs show relative cell numbers for each condition; line graphs show normalized response as described in legend to Figure 3A. p values are indicated as described in Figures 1 and 3. **(B).** Bpep and Dpep show similar potency in inhibiting survival of both replicating and non- or slowly-replicating cancer cell lines. Replicate cultures of T98G and MCF7 cells were pre-treated for 24 hours with 100 nM or 200 nM doxorubicin and then for another 6 days with indicated concentrations of Bpep and Dpep. Data are from one experiment carried out in triplicate. Left panel shows the ratios of cell numbers 6 days after pre-treatment (day 7) and cell numbers immediately following pre-treatment (day 1) for cultures treated with doxorubicin alone. p values are indicated as described in Figures 1 and 3. **(C).** Cell cycle distribution of HCT116 and T98G cells with and without 24 hours of treatment with 100 nM doxorubicin. Cultures were treated as indicated, stained with PI and subjected to flow cytometry analysis to reveal cell cycle distribution. Values on graph indicate percentage of cells in G1, S and G2M phases. Data represent results from one experiment. Comparable results achieved with repeat experiment for T98G cells.
