# Supplementary material for: Cell-Penetrating CEBPB and CEBPD Leucine Zipper Decoys as Broadly Acting Anti-Cancer Agents
Source: Cancers (Basel). 2021 May 20;13(10):2504. doi: 10.3390/cancers13102504 (PMC8161188; doi:10.3390/cancers13102504)
Supplement: Supplementary file 1 [file cancers-13-02504-s001.zip › cancers-1202275-supplementary 2/Supplementary Figure S7 + legend 5-21.pdf]

Supplementary Fig S7

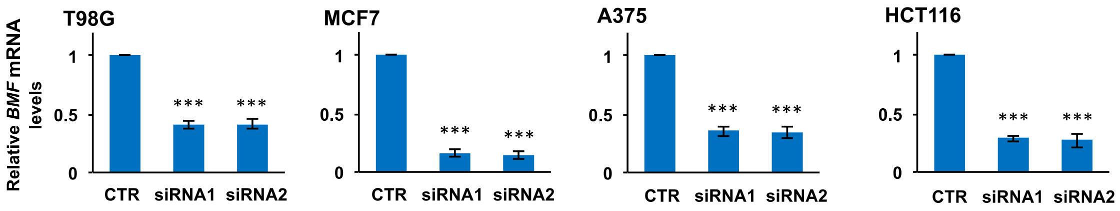

Supplementary Figure S7. **Knockdown of *BMF* by BMF-siRNA1 and BMF-siRNA2 in multiple cancer cell lines**. Replicate cultures were treated with siControl, BMF-siRNA1 and BMF-siRNA2 for 2 days and assessed by qPCR for relative levels of *BMF* transcripts. p values are indicated as described in Figure 1.
