# Supplementary material for: Cell-Penetrating CEBPB and CEBPD Leucine Zipper Decoys as Broadly Acting Anti-Cancer Agents
Source: Cancers (Basel). 2021 May 20;13(10):2504. doi: 10.3390/cancers13102504 (PMC8161188; doi:10.3390/cancers13102504)
Supplement: Supplementary file 1 [file cancers-13-02504-s001.zip › cancers-1202275-supplementary 2/Supplementary Figure S8 + legend 5-21.pdf]

Supplementary Fig S8

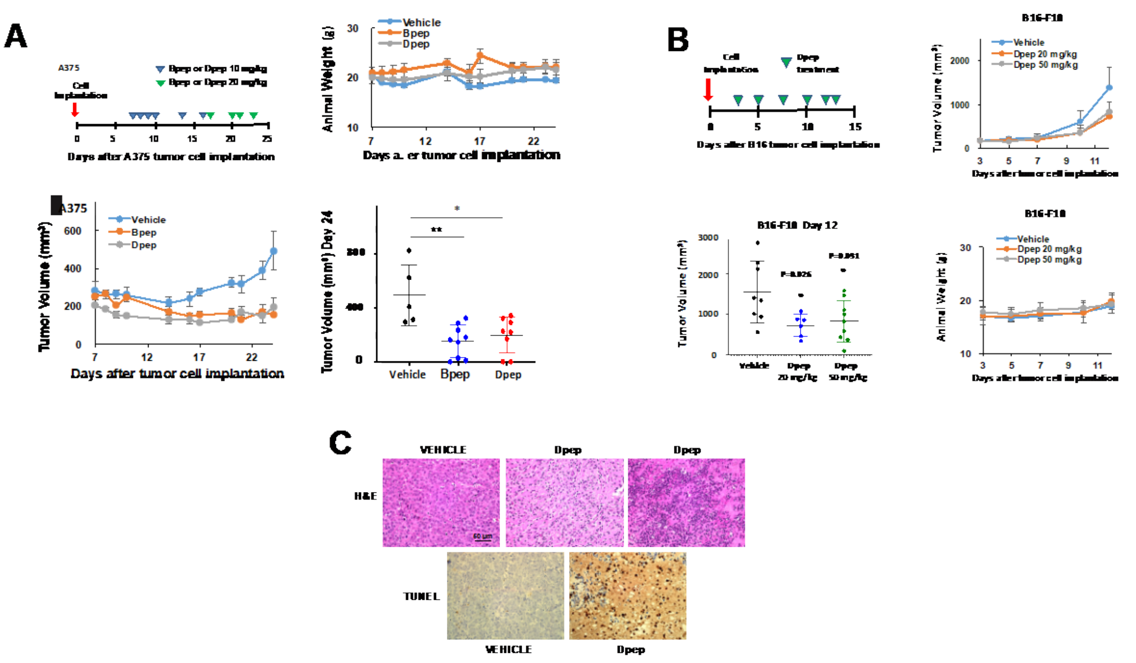

Supplementary Figure S8. ***In vivo* actions of Bpep and Dpep**. **(A).** Bpep and Dpep inhibit the growth of A375 melanoma cell xenograft tumors. A375 cells were subcutaneously implanted in the flanks of NCR nude mice. After tumors had formed, the animals were treated intraperitoneally with 10 or 20 mg/kg of Bpep, Dpep or vehicle as indicated in the scheme shown in the upper left panel. Tumor sizes were measured and volumes calculated on each day of injection. The lower left panel shows calculated mean tumor volumes vs time after tumor cell implantation. The study was ended on day 24 when one of the vehicle-treated animals showed morbid behavior. The lower right panel compares calculated individual and mean tumor volumes for vehicle, Bpep and Dpep treated animals on day 24. p values vs vehicle (N=5 tumors): Bpep 0.0028 (N=9 tumors); Dpep 0.0098 (N=8 tumors). The upper right panel shows mean animal weights vs time for each group of animals. **(B).** Dpep inhibits growth of B16-F10 melanoma cells in an allograft mouse model. B16-F10 cells were subcutaneously implanted in the flanks of C57BL/6 mice. After the tumors were established, the mice were treated intraperitoneally with 20 or 50 mg/kg of Dpep or vehicle as indicated in the scheme shown in the upper left panel. The upper right panel shows calculated mean tumor volumes for each group vs time up to the time (day 13) at which the first vehicle–treated animal reached the experimental endpoint when at least one tumor reached a calculated volume of > 1000 mm^3. The lower left panel compares calculated individual and mean tumor volumes for each group of animals on day 12. p values vs vehicle (N=8 tumors): 0.026, Dpep 20

mg/kg (N=7 tumors); 0.031, Dpep 50 mg/kg (N=9 tumors). The lower right panel shows mean animal weights vs time for each group. **(C).** High-power view showing degeneration and apoptosis of xenografted tumor cells in animals treated with Dpep. Subcutaneous A375 xenograft tumors were established in 4 mice. When the tumors reached approximately 300 mm^3, randomly chosen animals were treated with vehicle or 20 mg/kg of Dpep. The animals were retreated 2 days later and after an additional 2 days, the animals were sacrificed, and the tumors harvested for H&E and TUNEL staining. Top panels show H&E staining of sections from vehicle-treated animal B, tumor 2; Dpep-treated animal A, tumor 1 (center); and Dpep-treated animal B, tumor 2. Lower panels show TUNEL-stained sections from vehicle-treated animal A, tumor 1 and Dpep-treated animal A, tumor 1.
