# Supplementary material for: Cell-Penetrating CEBPB and CEBPD Leucine Zipper Decoys as Broadly Acting Anti-Cancer Agents
Source: Cancers (Basel). 2021 May 20;13(10):2504. doi: 10.3390/cancers13102504 (PMC8161188; doi:10.3390/cancers13102504)
Supplement: Supplementary file 1 [file cancers-13-02504-s001.zip › cancers-1202275-supplementary 2/Supplementary Figure S9.pdf]

Supplementary Figure S9

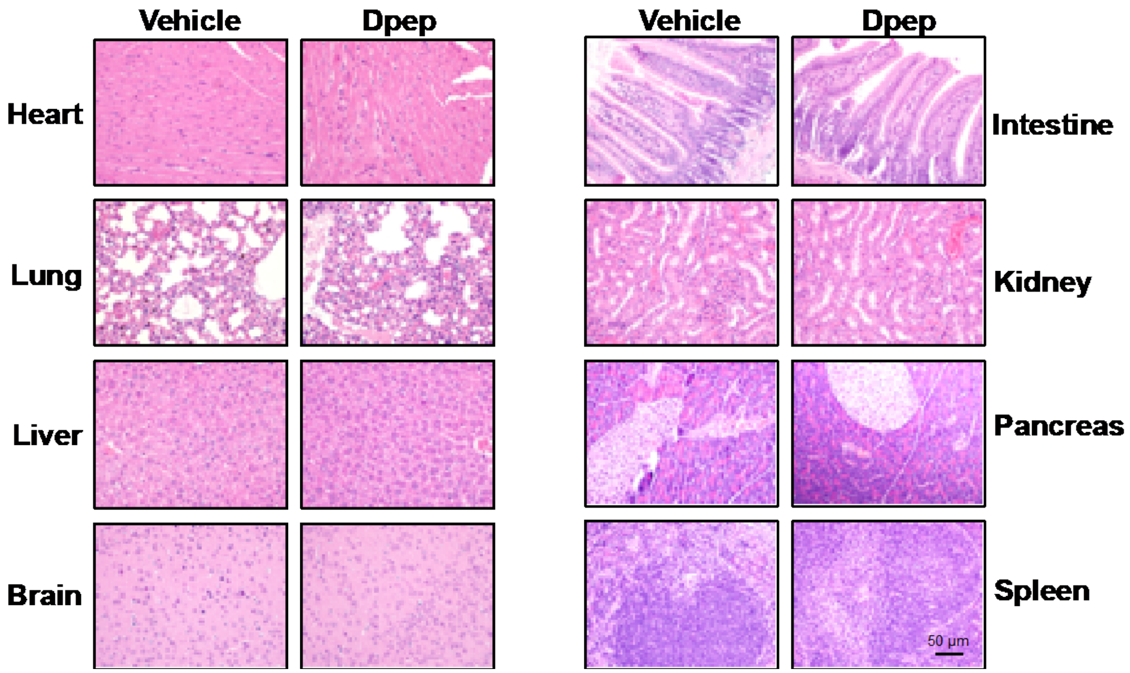

Supplementary Figure S9. **Dpep treatment does not affect the histological features of normal tissues**. Organs were removed at the endpoint of the experiment shown in Figure 6A in which animals were subjected to a full course of treatment with either Dpep or vehicle. Sections from the fixed tissues were stained with H&E. Panels show examples of sections of tissue from vehicle and Dpep-treated animals.
