# Supplementary material for: Cell-Penetrating CEBPB and CEBPD Leucine Zipper Decoys as Broadly Acting Anti-Cancer Agents
Source: Cancers (Basel). 2021 May 20;13(10):2504. doi: 10.3390/cancers13102504 (PMC8161188; doi:10.3390/cancers13102504)
Supplement: Supplementary file 1 [file cancers-13-02504-s001.zip › cancers-1202275-supplementary 2/Supplementry Figure S1 + legend 5-21.pdf]

Supplementary Fig. S1

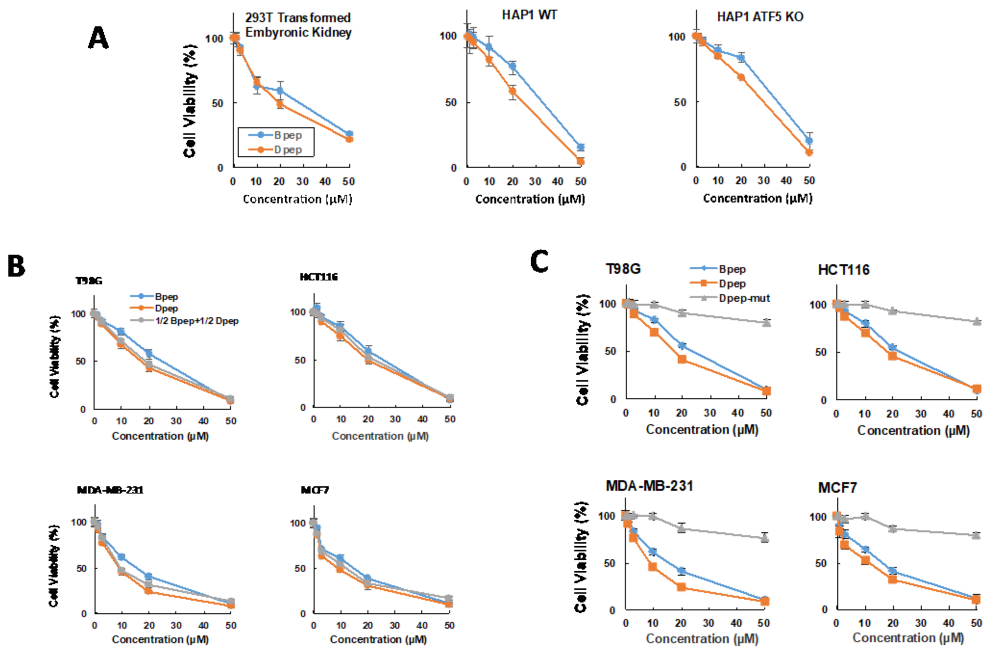

Supplementary Figure S1. **Bpep and Dpep activities on cultured tumor cells**. **(A)**. Bpep and Dpep suppress growth/survival of 293T transformed embryonic kidney cells and of wild type (WT) and ATF5KO HAP1 myelogenous leukemia cells. Replicate cultures of cells were treated with the indicated concentrations of peptides for 6 days and assessed for cell number. Data are from one of two independent experiments, each in triplicate, with comparable results. **(B).** Bpep and Dpep show additive effects of growth/survival of multiple cancer cell lines when used in combination. Replicate cultures were treated for 6 days with Bpep and Dpep at the indicated concentrations or in combination at a 1:1 molar ratio to reach the indicated final concentrations. Data are from one of two independent experiments, each in triplicate, with comparable results. **(C).** Dpep potency requires leucine zipper heptad repeat leucines. Replicate cultures of indicated cancer cell lines were treated with indicated concentrations of Bpep, Dpep or Dpep-mut and assessed 6 days later for cell number. Data are from one of two independent experiments, each in triplicate, with comparable results.
